# The emerging roles of ITM2A: At the molecular crossroads of development and pathogenesis

**DOI:** 10.1016/j.isci.2026.116657

**Published:** 2026-07-14

**Authors:** Xiaoling Deng, Yifang Wang, Lan Yang, Kangli Wu, Wenying Qin, Declan William Ali, Marek Michalak, Jingfeng Tang, Cefan Zhou, Xing-Zhen Chen

**Affiliations:** 1 National “111” Center for Cellular Regulation and Molecular Pharmaceutics, Key Laboratory of Fermentation Engineering (Ministry of Education), Cooperative Innovation Center of Industrial Fermentation (Ministry of Education & Hubei Province), Hubei Key Laboratory of Industrial Microbiology, Hubei University of Technology, Wuhan, China; 2 Cooperative Innovation Center of Industrial Fermentation (Ministry of Education & Hubei Province), Hubei Key Laboratory of Industrial Microbiology, Hubei University of Technology, Wuhan, China; 3 Department of Biological Sciences, University of Alberta, Edmonton, AB, Canada; 4 Department of Biochemistry, University of Alberta, Edmonton, AB, Canada; 5 Membrane Protein Disease Research Group, Department of Physiology, Faculty of Medicine and Dentistry, University of Alberta, Edmonton, AB, Canada

**Keywords:** ITM2A, regulation and molecular mechanisms, development, pathogenesis, promising target, potential biomarker

## Abstract

Integral membrane protein 2A (ITM2A) is a type II transmembrane glycoprotein belonging to the BRICHOS superfamily. It primarily regulates organismal development and homeostasis and exhibits tumor-suppressive functions. Its expression is precisely regulated by a multidimensional network involving transcription factors, epigenetic modifications, and environmental signals. Within cellular signaling networks, ITM2A modulates multiple key pathways, including BMP, JAK/STAT, ERK, Hedgehog, and PKA-CREB. ITM2A is essential for the differentiation and functional maturation of various tissues, such as cartilage, bone, and muscle. Dysregulation of ITM2A function or expression is closely associated with malignancies, thyroid disorders, and acute transplant rejection. This review systematically summarizes the complex regulatory mechanisms of ITM2A in growth, development, and disease, with a particular focus on skeletal development and tumorigenesis, aiming to provide a theoretical basis for its potential use as a biomarker for development and disease diagnosis/treatment, as well as for the development of ITM2A-targeted therapeutics.

## Graphical abstract

**Figure:**
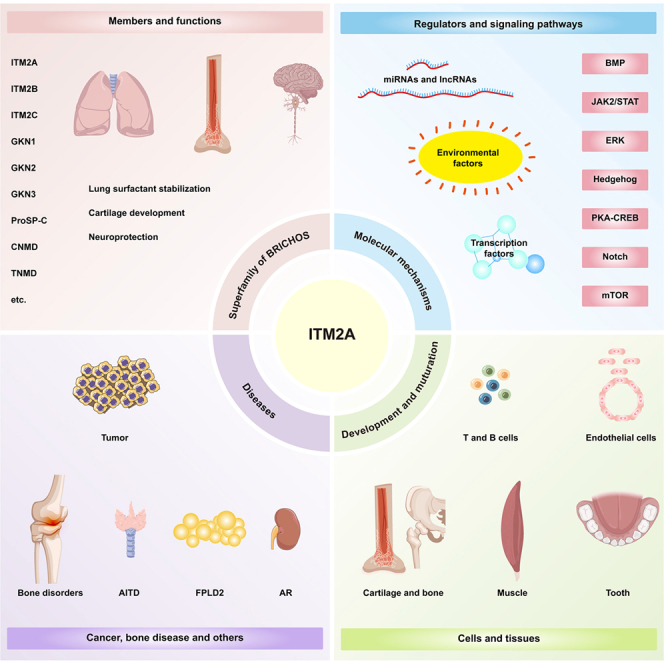

## Teaser

Biological sciences; Molecular biology

## Introduction

The BRICHOS domain is a conserved functional module of approximately 100 amino acid residues. The name of BRICHOS derives from the initials of the three key precursor proteins in which it was first identified: ITM2B (BRI2), associated with hereditary dementia; chondromodulin-I (CHM-I), a cartilage-specific angiogenesis inhibitor; and prosurfactant protein C (proSP-C), mutations of which cause familial interstitial lung disease.^1^^,^^2^^,^^3^ This nomenclature not only reflects the domain’s discovery history but also underscores the fundamental role of the BRICHOS superfamily in maintaining protein homeostasis across diverse tissues. Despite their varied functions, its members share a conserved molecular module of the BRICHOS domain. The core role of the BRICHOS domain is to function as an intramolecular chaperone. It is primarily responsible for preventing the formation of toxic amyloid fibrillar aggregates in host proteins, as well as ensuring their proper protein folding and maturation. The BRICHOS superfamily displays both structural complexity and functional diversity, with established associations with numerous diseases, including neurodegenerative disorders, respiratory distress, cancer, and even conditions such as SARS-CoV-2 infection.^2^^,^^4^

ITM2A belongs to the ITM2 (Type II integral membrane protein) family, which represents one of the core branches within the BRICHOS superfamily. It has two closely related homologs: ITM2B and ITM2C. ITM2A is a single-pass transmembrane protein with an intracellular N-terminus and an extracellular C-terminus. It is characterized as an unstable, hydrophilic, non-secretory protein primarily localized to the cell membrane.^5^ This structural topology confers distinct biological roles in cellular signal sensing, tissue differentiation, and disease regulation. ITM2A was first isolated and documented through the screening of cDNA libraries derived from the mandibular articular organ cultures of prenatal mice.^6^ In humans, it exhibits differential expression in multiple tissues, including the ovary, thyroid, adipose tissue, and heart.

The expression of ITM2A is tightly regulated by multiple mechanisms, including environmental stimuli, miRNAs, transcription factors, and signaling pathways. External factors such as lipopolysaccharide (LPS), interferon-gamma (IFN-γ), and manganese chloride (MnCl_2_) have been shown to modulate ITM2A expression.^7^^,^^8^ Additionally, binding by miRNAs such as miR-1 and miR-296 inhibits its expression.^9^^,^^10^ ITM2A also acts as a downstream effector of the BMP and PKA-CREB signaling pathways.^11^^,^^12^ Functional studies indicate that ITM2A plays a vital role in biological processes, including autophagy and cartilage development, through the regulation of pathways including mammalian target of rapamycin (mTOR), BMP, and Hedgehog (Hh).^11^^,^^13^^,^^14^ Moreover, substantial evidence links the dysregulation of ITM2A to various human diseases. Abnormal expression or function of ITM2A is associated with thyroid disorders, acute transplant rejection, and the onset, progression, and clinical prognosis of malignant tumors, including breast, ovarian, and cervical cancers.^8^^,^^13^^,^^14^^,^^15^^,^^16^

This paper summarizes the functions of BRICHOS superfamily proteins and provides a systematic overview of recent advances in ITM2A research. Its primary aim is to elucidate the complex regulatory networks through which ITM2A influences organismal growth, development, and disease progression. By analyzing the unique functions of ITM2A in depth, this review seeks to establish a theoretical and experimental foundation for its potential applications in disease diagnosis, prognosis, and therapeutic intervention.

## Methodology

We conducted a comprehensive literature search using the PubMed database with the keywords “ITM2A,” “BRI1,” and “BRICHOS.” The search included all studies published up to 2025, excluding preprints. Our review focused on fundamental information about ITM2A, its molecular mechanisms, and its associations with human diseases.

## Superfamily of proteins containing a BRICHOS domain

**Figure 1:**
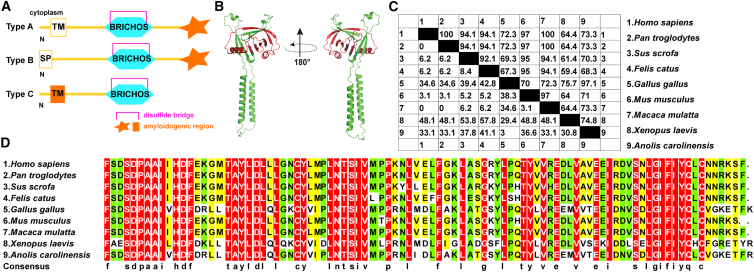
Schematic diagram of the BRICHOS proprotein structure, and structural prediction of ITM2A and a sequence alignment of its BRICHOS domain (A) Shows the three structural types of the BRICHOS proprotein, including the transmembrane domain (TM), signal peptide (SP), BRICHOS domain, conserved disulfide bonds, and the amylated region. (B) Shows the structural prediction of ITM2A, with the red region representing the BRICHOS domain. (C) Shows a homology analysis of the BRICHOS domain in ITM2A across different species. (D) Shows a sequence analysis of the BRICHOS domain in ITM2A across different species.

In 2002, the Sánchez-Pulido team used bioinformatics tools to identify the BRICHOS domain, which consists of around 100 amino acids. This study marked the first time the BRICHOS domain was linked to three major clinical conditions: dementia, respiratory distress, and cancer.^1^ To date, the BRICHOS domain has been identified in 12–14 proteins, in proteins with known functions including ITM2A, ITM2B, ITM2C, GKN1, GKN2, proSP-C, CHM-I, and tenomodulin (TNMD)^2^^,^^17^ (Table 1). The common structural organization of BRICHOS superfamily precursor proteins can be broadly classified into three categories, as illustrated in Figure 1A.^4^

**Table 1:** Basic information and functions of proteins containing a BRICHOS domain

Protein	Synonym	Gene location	Length	Expression	Function/disease	Reference
ITM2A	BRI1, ITM2, E25A	Xq21.1	263	ovary, thyroid, fat, lymph node, heart, brain	bone and chondrogenic development, muscle differentiation, the regulation of autophagy, and tumour suppression	^11^^,^^12^^,^^14^^,^^18^^,^^19^^,^^20^^,^^21^^,^^22^^,^^23^
ITM2B	BRI2, E25B	13q14.3	266	kidney, placenta, thyroid, fat, endometrium	as a molecular chaperone, related to neuronal dysfunction and dementia, male reproduction, and tumour suppression	^1^^,^^24^^,^^25^^,^^26^^,^^27^^,^^28^^,^^29^^,^^30^
ITM2C	BRI3, ITM3, E25C	chromosome 2	267	brain, colon	as a molecular chaperone, related to AD	^29^,^31^^,^^29^^,^^32^^,^^33^^,^^34^
GKN1	CA11, FOV, AMP-18	chromosome 2p13.3	185	stomach	plays a role in gastric function and gastric mucosal protection	^35^^,^^36^^,^^37^^,^^38^
GKN2	GDDR, TFIZ1	chromosome 2p13.3	184	stomach, lung	may be a tumour suppressor	^36^^,^^39^^,^^40^^,^^41^^,^^42^
proSP-C	SFTPC	8p21.3	165	lung	as a molecular chaperone, plays a key role in the synthesis of pulmonary surfactant	^43^^,^^44^^,^^45^^,^^46^^,^^47^^,^^48^
CNMD	Chm-1, Chm-I, Lect-1	chromosome 13q14-21	334	thyroid, salivary gland, testis, brain, spleen	regulation of angiogenesis, cartilage development, and homeostasis	^49^^,^^50^^,^^51^^,^^52^^,^^53^^,^^54^^,^^55^^,^^56^^,^^57^
TNMD	CHM1L, TEM	Xq22.1	317	fat, ovary, placenta, prostate, appendix	maintain collagen tissue homeostasis and inhibit pathological angiogenesis	^58^^,^^59^^,^^60^^,^^61^^,^^62^

### ITM2 family

The ITM2 family is highly conserved and includes ITM2A (BRI1), ITM2B (BRI2), and ITM2C (BRI3). All three are integral type II transmembrane glycoproteins, with the C-terminal region oriented extracellularly.^31^ Early studies mapped *ITM2A* to the broader chromosomal region Xq13.3–q21.2^63^; however, with the refinement of the human genome map, its precise location has been identified as Xq21.1.^18^ ITM2A is implicated in multiple biological processes, including chondrogenic differentiation,^19^^,^^20^ muscle differentiation,^21^ bone healing,^11^ the regulation of autophagy,^12^ and tumor suppression.^14^ It is also highly expressed in cerebral microvascular endothelial cells, suggesting potential as a target for brain-directed drug delivery,^22^^,^^23^ although this requires further validation. The predicted protein structure and sequence alignment of the BRICHOS domain in ITM2A are shown in Figures 1B–1D. ITM2B exhibits anti-amyloid aggregation chaperone activity. Extensive studies indicate that mutations in ITM2B produce longer mature peptides during post-translational processing, which aggregate into amyloid fibrils. These fibrillar deposits accumulate in the brain, leading to cognitive impairment and memory loss.^1^^,^^24^^,^^25^ ITM2B is also associated with male reproduction^26^ and tumor suppression.^27^^,^^28^ ITM2C is primarily brain-specific and prone to forming large protein aggregates.^31^^,^^32^ It functions mainly within cells or near the cell surface, making it a potential therapeutic target for Alzheimer’s disease (AD).^29^^,^^33^^,^^34^

### Gastrokine family

The gastrokines are classified into three families: GKN1, GKN2, and GKN3. Among these, GKN1 and GKN2 have been implicated in gastric cancer.^17^ The *GKN1* gene shows strong evolutionary conservation in multiple species. Its mRNA is abundantly expressed in the human stomach, the antrum, and cardiac tissue, but is absent in both intestinal and diffuse gastric carcinomas, suggesting a critical role in maintaining gastric mucosal integrity.^35^ *GKN2* is associated with gastritis and gastric cancer.^39^^,^^40^^,^^41^ It is undetectable in 85% of diffuse gastric cancers and 54% of intestinal cancers, with loss of expression significantly correlating with poor patient prognosis.^40^^,^^42^ In contrast to *GKN1* and *GKN2*, *GKN3* appears to be a novel gastric gene with unique functions that is linked to inflammation. In humans, *GKN3* is a nonfunctional pseudogene. In mice, its mRNA is nearly silent in healthy gastric mucosa but is significantly upregulated in TxA23 transgenic mice and in models of Helicobacter pylori infection.^64^^,^^65^

### ProSP-C

Surfactant protein C (SP-C) has high hydrophobicity and amyloidogenic potential.^66^^,^^67^^,^^68^^,^^69^ To safely synthesize this aggregation-prone peptide, cells first produce a larger precursor protein, proSP-C. This precursor contains the BRICHOS domain, which inhibits the formation of the amyloid-like domain of SP-C during biosynthesis.^43^^,^^44^^,^^45^^,^^46^ Moreover, it has been demonstrated to inhibit the fibrillation of various pathogenic amyloids, including Aβ and α-Synuclein.^47^^,^^48^ Therefore, ProSP-C plays a crucial role in protein quality control, surfactant metabolism dysregulation, and related pulmonary diseases.

### CNMD

Human chondromodulin (CNMD) was initially isolated from fetal bovine cartilage.^49^^,^^50^ Its transcripts are enriched in healthy and developing avascular cartilage as well as in cardiac valve endothelium.^50^^,^^51^ Functionally, CNMD is involved in regulating angiogenesis, cartilage development, and tissue homeostasis.^50^^,^^52^^,^^53^ Clinically, dysregulated expression or functional abnormalities of CNMD have been closely associated with the onset and progression of osteoarthritis (OA),^54^ infective endocarditis,^55^ breast cancer,^56^ and malignant bone tumors.^57^ These findings suggest that CNMD may serve as a therapeutic target and a key regulatory node within the vascular-cartilage-bone axis.

### TNMD

TNMD is a tendon- and ligament-specific marker as well as an endogenous anti-angiogenic molecule. Its expression is highly restricted to tissues with low vascular density, including ligaments, tendons, and ocular tissues.^58^^,^^59^ Functional loss or abnormal expression of TNMD has been associated with intervertebral disc (IVD) degeneration,^60^ AD,^61^ age-related macular degeneration (AMD),^62^ adipocyte accumulation, and fibrovascular scarring. These observations suggest that TNMD functions as a critical molecular regulator, maintaining collagenous tissue homeostasis and suppressing pathological angiogenesis.

In summary, the primary function of the BRICHOS domain is molecular chaperone activity, a role that has been experimentally validated in many family members. Proteins within this superfamily participate in diverse physiological and pathological processes, including cartilage development,^19^^,^^49^^,^^51^ pulmonary surfactant homeostasis,^43^^,^^45^^,^^46^ neuroprotection,^28^^,^^32^^,^^34^ and tumor suppression.^14^^,^^35^^,^^36^^,^^41^ While earlier studies have demonstrated clear molecular chaperone functions in certain members such as ITM2B, ITM2C, and proSP-C,^4^^,^^17^^,^^29^^,^^47^^,^^48^^,^^70^ bioinformatics predictions and high-throughput screens indicate the existence of additional, uncharacterized members with potentially unique structural and functional roles. Notably, within the ITM2 subfamily, all members except ITM2A, namely ITM2B and ITM2C, have confirmed molecular chaperone activity. Interestingly, motopsin, a protein associated with intellectual disability, has been shown to interact specifically with the BRICHOS domain of ITM2A,^22^ suggesting that ITM2A may possess chaperone functions in the nervous system. However, the precise role of ITM2A remains unclear, implying possible functional differentiation or unique mechanisms of action. Elucidating the molecular functions of ITM2A will enhance our understanding of its involvement in chondrogenesis, muscle formation, and T cell maturation, and may provide a theoretical basis for its potential clinical relevance in skeletal disorders, tumorigenesis, and neurological diseases associated with the BRICHOS domain.

## Regulation and molecular mechanisms of ITM2A

### Regulators of ITM2A

**Figure 2:**
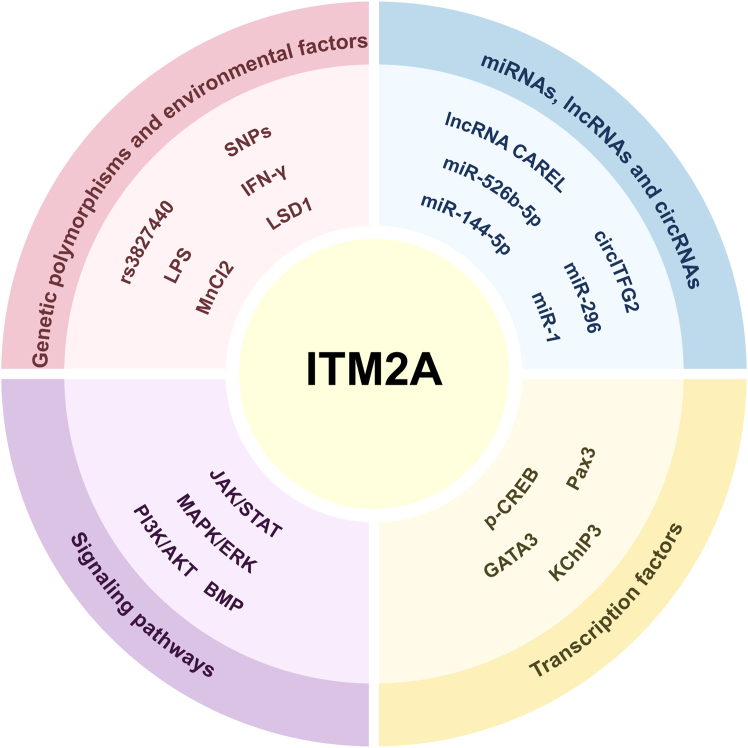
The main factors involved in the regulation of ITM2A Genetic polymorphisms and environmental factors include rs3827440, SNPs, LPS, IFN-γ, MnCl_2_, and LSD1; miRNAs, lncRNAs, and circRNAs comprise lncRNA CAREL, circITFG2, miR-144-5p, miR-296, miR-526b-5p, and miR-1; transcription factors include *p*-CREB, GATA3, Pax3, and KChIP3; Signaling pathways include JAK/STAT, MAPK/ERK, PI3K/AKT, and BMP.

Current research indicates that the expression and activity of ITM2A are precisely regulated at multiple levels, including genetic, epigenetic, transcriptional, environmental factors, and signaling pathways. The primary upstream regulatory factors include the following categories: genetic polymorphisms, environmental factors, miRNAs, lncRNAs, circular RNAs (circRNAs), transcriptional regulation, and signaling pathways (Figure 2).

Research has identified rs3827440 and its linked single nucleotide polymorphisms (SNPs) as likely pathogenic variants within the Xq21.1 region, with their genotype dosage significantly correlating with baseline ITM2A expression levels in both peripheral blood mononuclear cells (PBMCs) and monocytes.^8^ Notably, upon stimulation with bacterial LPS or the viral mimic IFN-γ, differences in ITM2A expression between individuals with different genotypes were markedly amplified in monocytes, suggesting that environmental factors such as infections can modulate ITM2A expression via interactions with genetic susceptibility loci.^8^ In a Mn^2+^ stimulation model, treatment with MnCl_2_ significantly downregulated *ITM2A* mRNA in mouse peritoneal macrophages, indicating that metal ions may indirectly regulate the expression of ITM2A through epigenetic mechanisms or signaling pathways that remain to be elucidated.^7^ Furthermore, *Prrx1*-lineage cells, which are key contributors to fracture healing, express ITM2A as a specific surface marker in *Prrx1*-positive periosteal stem cells. The lysine-specific demethylase 1A (LSD1) acts as a critical epigenetic regulator during fracture repair and injury healing. Deficiency of LSD1 in *Prrx1*-lineage cells impairs fracture healing, suggesting that LSD1 may modulate *ITM2A* expression by modifying chromatin structure.^11^^,^^71^

Current research on the regulatory roles of miRNAs, long non-coding RNAs (lncRNAs) and circRNAs in ITM2A expression remains limited. Certain miRNAs primarily target the 3′-untranslated region (3′-UTR) of mRNAs, inducing degradation or translational repression to achieve post-transcriptional regulation.^72^ Some lncRNAs can competitively bind to miRNAs, thereby indirectly modulating the expression of target genes.^73^^,^^74^^,^^75^ CircRNAs most commonly function as miRNA sponges; they contain multiple miRNA-binding sites that prevent miRNAs from interacting with their target mRNAs, indirectly upregulating gene expression.^76^ For example, miR-1, a skeletal muscle-specific miRNA, has been shown in mouse models to downregulate *ITM2A* mRNA levels.^9^ In a study examining exercise-induced changes in the miRNA-mRNA network in the rat hippocampus, miR-144-5p exhibited a negative correlation with *ITM2A* expression.^77^ In mouse models and cardiomyocytes, lncRNA CAREL reduces the inhibitory effect of miR-296 on ITM2A by competitively binding to miR-296, thereby regulating cardiomyocyte proliferation and regeneration after injury.^10^ In lung squamous cell carcinoma (LUSC), circITFG2 overexpression upregulates ITM2A expression by competitively binding to miR-526b-5p.^78^

Transcription factors can bind specifically to DNA *cis*-regulatory elements, such as promoters, enhancers, and silencers, thereby quantitatively or qualitatively altering the transcription of target genes.^79^ For *ITM2A*, cAMP agonists or PKA overexpression significantly enhance promoter activity, whereas mutation of the cAMP response element (CRE) site or knockdown of CREB via siRNA reduces its expression. Chromatin immunoprecipitation (ChIP) experiments confirmed that phosphorylated CREB (*p*-CREB) directly occupies the *ITM2A* promoter.^12^ Similarly, ChIP-qPCR revealed that GATA3 binds to the *ITM2A* promoter and drives transcriptional activation in thymus-dependent T cells.^80^ In *GATA3*-deficient mice, ITM2A expression in the thymus is significantly reduced, resulting in partial developmental blockade of MHC-I-restricted thymocytes.^80^ Reporter gene assays and embryonic *in situ* hybridization further demonstrated that Pax3 directly activates *ITM2A* transcription, and the loss of Pax3 markedly reduced ITM2A expression in embryos.^21^^,^^81^ Additionally, Kv Channel-Interacting Protein 3 (KChIP3), a member of the neuronal calcium sensor (NCS) family with both calcium-sensing and nuclear transcription-inhibitory functions, was identified as an upstream regulator of ITM2A. Comparative RNA sequencing (RNA-seq) analysis of forebrain cortex from wild-type and *Kcnip3*^−/−^ rats revealed reduced *ITM2A* mRNA in the absence of *KChIP3*, supporting its role in transcriptional regulation.^82^

The expression of ITM2A is precisely regulated by multiple signaling pathways. In skeletal biology, the BMP signaling pathway is crucial for the regulation of ITM2A. Studies have shown that BMP signaling is highly enriched in *ITM2A*^+^ periosteal skeletal stem cells (P-SSCs). Specific knockout of the *Bmp2* gene in murine *ITM2A*^+^ P-SSCs impairs fracture healing, directly demonstrating that BMP signaling acts as a key upstream regulator of *ITM2A*^+^ P-SSCs during fracture repair, likely by directly or indirectly regulating ITM2A expression or maintaining the stem cell population.^11^ In oncology, ITM2A expression is downregulated in drug-resistant chronic myeloid leukemia (CML) cells. Overexpression of ITM2A reduces *p*-ERK levels and restores imatinib sensitivity, indicating a negative feedback relationship between ERK activity and ITM2A expression.^83^ In prostate cancer, CXCR1, a G protein-coupled receptor (GPCR) and high-affinity receptor for IL-8, activates signaling cascades including JAK/STAT, mitogen-activated protein kinase (MAPK)/ERK, and PI3K/AKT. Overexpression of CXCR1 in the MDA-PCa-2b androgen-dependent prostate cancer cell line significantly increases ITM2A expression, leading to the inhibition of tumor growth.^84^ In bladder cancer cells and mouse models, ITM2A downregulates the STAT3 signaling pathway, while STAT3 activity may conversely modulate ITM2A levels, suggesting a mutually restraining negative feedback loop. Activated STAT3 may suppress ITM2A transcription, although this mechanism requires further experimental validation.^85^

### Signaling pathways of ITM2A

**Figure 3:**
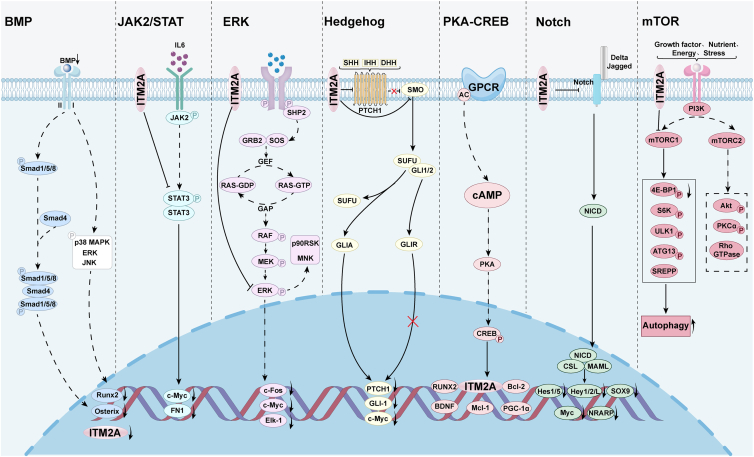
Major signaling pathways mediated by ITM2A and their regulatory mechanisms The BMP signaling directly or indirectly regulates ITM2A expression; ITM2A inhibits STAT3 activation, thereby suppressing downstream target gene activation; ITM2A induces cell-cycle arrest by reducing phosphorylation levels in the ERK signaling pathway; ITM2A acts as a negative regulator of both canonical and non-canonical Hh signaling; PKA-CREB positively regulates ITM2A expression; ITM2A downregulates components of Notch signaling, thereby suppressing the expression of downstream target genes; ITM2A inhibits mTOR activity, thereby promoting autophagy.

ITM2A is an important membrane protein that participates in and regulates multiple signaling pathways (Figure 3), playing significant roles in various biological processes and disease contexts. Its functions include maintaining SSC activity, promoting fracture healing, and modulating tumorigenesis, cancer progression, and drug resistance.

### BMP signaling pathway

Within the BMP signaling network, ligands closely related to bone repair and vascularization include BMP-2, BMP-4, BMP-6, BMP-7, BMP-9, and BMP-10. These ligands form hetero-oligomeric complexes with type I (ALK1/2/3/6) and type II serine/threonine kinase receptors on the cell membrane, with type I receptors acting as the primary switches for signal transduction. Notably, ALK1 is predominantly enriched in endothelial cells, whereas ALK2, ALK3, and ALK6 are primarily expressed on the membranes of osteogenically differentiated cells. Type II receptors, including BMPR2 and ActR2A/2B, mediate ligand binding and phosphorylate type I receptors. The BMP pathway transduces signals mainly through canonical Smad-dependent mechanisms and non-Smad pathways such as p38 MAPK, ERK, and JNK. Upon ligand binding, type I receptors phosphorylate intracellular Smads (primarily Smad1/5/8), which then complex with Smad4 and translocate to the nucleus. This process activates the transcription of target genes, including Runx2 and Osterix, thereby regulating osteogenic differentiation and cellular function.^86^^,^^87^^,^^88^^,^^89^

In skeletal biology, the BMP signaling pathway exhibits particularly close associations with ITM2A. Research has revealed that the BMP signaling pathway is highly enriched in *ITM2A*^+^ P-SSCs. Conditional knockout of the *BMP2* gene in mouse *ITM2A*-lineage cells resulted in impaired fracture healing, directly demonstrating that BMP signaling serves as a critical upstream regulator of *ITM2A*^+^ cells during fracture repair. These findings suggest that BMP signaling likely regulates ITM2A expression either directly or indirectly, or helps maintain the functional integrity of this stem cell population.^11^

### JAK2/STAT signaling pathway

The JAK2/STAT pathway is one of the most representative branches of the JAK/STAT signaling family. Its defining feature is the direct transmission of signals from cell membrane receptors to the nucleus, thereby initiating gene transcription without the involvement of second messengers. Activation of this pathway occurs in four steps. First, cytokines (such as IL-6, IL-3, IFN-γ, EPO, TPO, and GM-CSF) or growth factors bind to transmembrane receptors, inducing receptor dimerization or multimerization. Second, receptor dimerization brings associated JAK2 molecules into close proximity, enabling their *trans*-phosphorylation and full activation of kinase activity. Activated JAK2 then phosphorylates tyrosine residues on the receptor’s intracellular domain, creating docking sites for STAT proteins. Third, cytoplasmic STAT proteins recognize and bind to these phosphorylated tyrosine residues via their SH2 domains. JAK2 subsequently phosphorylates specific tyrosine residues at the C-terminus of the STAT proteins, leading to their dissociation from the receptor and formation of homo- or heterodimers. Fourth, STAT dimers translocate into the nucleus through nuclear pores, where they bind to gamma-interferon activation site/interferon-sensitive response element (GAS/ISRE) sequences within target gene promoters. Additional transcriptional cofactors are recruited to initiate gene transcription of downstream effectors (e.g., Bcl-xL, c-Myc, Cyclin D1, SOCS3, and p21), thereby regulating biological processes such as proliferation, survival, differentiation, and inflammation.^90^^,^^91^^,^^92^^,^^93^

ITM2A acts as a tumor suppressor in bladder cancer. RNA-seq analysis revealed significant enrichment of JAK/STAT, ErbB, and MAPK signaling pathways in bladder cancer cells overexpressing ITM2A. Functional experiments further demonstrated that ITM2A inhibits IL-6-mediated STAT3 phosphorylation, leading to the downregulation of downstream target genes, including c-Myc and FN1, and thereby suppressing the malignant phenotype of bladder cancer cells. In a xenograft mouse model, tumors overexpressing ITM2A exhibited slower growth, with markedly reduced expression levels of c-Myc and FN1 in tumor tissues.^85^

### ERK signaling pathway

The ERK signaling axis is the best-known branch of the MAPK/ERK pathway. It transduces extracellular stimuli, including growth factors, cytokines, and mechanical stress, into nuclear transcriptional responses, governing essential biological processes such as proliferation, differentiation, survival, and tissue regeneration.^94^^,^^95^^,^^96^ The ERK signaling pathway is organized around a highly conserved protein kinase cascade. Growth factors, such as EGF, bind to receptor tyrosine kinases (RTKs, e.g., EGFR, PDGFR, FGFR), thereby initiating the classic activation sequence that ignites the ERK cascade. This process induces receptor dimerization and autophosphorylation. This is followed by the recruitment of adaptor proteins and activation of RAS (RAS-GTP). Activated RAS recruits RAF kinase to the plasma membrane, where it is activated and subsequently phosphorylates MEK on serine/threonine residues. MEK then dually phosphorylates ERK at T202 and Y204, resulting in full activation of ERK. Activated ERK mediates its biological effects through two primary mechanisms: (1) phosphorylation of cytoplasmic substrates, including p90RSK (regulating translation and cell survival) and MNK (regulating translation), and (2) nuclear translocation, where it phosphorylates transcription factors such as c-Fos, c-Myc, and Elk-1. These events regulate the expression of genes critical for cell cycle progression, proliferation, and differentiation.^97^^,^^98^

The ERK signaling pathway is one of the most extensively studied cascades in cancer research. In CML, ITM2A has been shown to interact with this pathway. Specifically, upregulation of ITM2A in imatinib-resistant CML cell lines reduces ERK phosphorylation, thereby increasing the sensitivity of resistant cells to the targeted therapy imatinib and inducing G2-phase cell-cycle arrest.^83^

### Hedgehog signaling pathway

The Hh signaling axis regulates embryonic development, adult stem cell homeostasis, and tissue regeneration through a three-level switch mechanism involving membrane receptors, cilia, and transcription factors. Dysregulated Hh signaling, however, is closely associated with the pathogenesis of various diseases, particularly tumorigenesis and cancer progression. The Hh signaling pathway is categorized as either canonical or non-canonical, based on its dependence on key signaling components and downstream molecular mechanisms. The canonical pathway follows a linear cascade that primarily regulates gene transcription and plays a key role in cell fate decisions, including proliferation, differentiation and apoptosis. Dysregulation typically arises from mutations in key components, such as receptors, kinases, or transcription factors, and is critically implicated in tumorigenesis. In contrast, the non-canonical pathway exhibits a highly divergent and complex architecture, often involving non-linear signal integration and crosstalk with other pathways. It primarily mediates rapid cellular responses, such as cytoskeletal reorganization, cell migration, and invasion, and is commonly activated by ligand overexpression or sustained receptor stimulation rather than genetic mutations. This pathway is especially important in promoting tumor metastasis, tissue invasion, and fibrotic processes.^99^^,^^100^^,^^101^ Hh pathway activation follows the classical “inhibition-release-activation” model. In the absence of Hh ligands, Sonic Hh (Shh), Indian Hh (Ihh), or Desert Hh (Dhh), the pathway remains inactive. Binding of Hh ligands triggers internalization and degradation of PTCH1, relieving its inhibition of Smoothened (SMO). This inhibition allows SMO to undergo conformational changes and phosphorylation, followed by translocation into the primary cilium. Activated SMO then suppresses the function of suppressor of fused (SUFU), which normally promotes the proteolytic processing of GLI proteins into transcriptional repressors. As a result, full-length GLI proteins, primarily GLI1 and GLI2, are stabilized and processed into their transcriptional activator forms (GLI-A). These GLI-A proteins translocate into the nucleus and initiate the transcription of downstream target genes, including Hh pathway components such as PTCH1and GLI1, as well as genes that regulate cell cycle progression and other critical processes.^99^^,^^100^^,^^101^

The Hh receptor PTCH1 and ITM2A independently inhibit autophagic flux, primarily by impairing autolysosome formation rather than affecting early autophagosome synthesis. Although ITM2A can interact with PTCH1, both proteins independently inhibit autophagic flux. Knockdown of ITM2A upregulates PTCH1 expression, enhancing PTCH1-mediated blockade of autophagic flux, whereas overexpression of ITM2A reduces PTCH1 protein levels. Concurrently, ITM2A overexpression suppresses the Gli-luciferase activation induced by the oncogenic SMO mutant (SMO-M2), Shh, Gli1, and Gli2. Collectively, these findings demonstrate that ITM2A exerts a negative regulation of both the canonical and non-canonical Hh signaling.^102^

### PKA-CREB signaling pathway

The PKA-CREB signaling pathway is a classical and highly conserved signal transduction mechanism that plays a central role in cellular responses to extracellular stimuli, including hormones and neurotransmitters. It regulates gene expression, thereby influencing essential biological processes such as cell growth, differentiation, survival, metabolism, and neural plasticity.^103^^,^^104^ The pathway is initiated when extracellular first messengers bind to GPCRs, activating adenylate cyclase (AC). This enzyme catalyzes the conversion of ATP to cAMP, rapidly increasing intracellular cAMP levels. Elevated cAMP triggers the dissociation of the regulatory subunits from the catalytic subunits of PKA. The free catalytic subunits then translocate into the nucleus and phosphorylate the transcription factor CREB at S133. Phosphorylated CREB binds to CREs in the promoter region of target genes, recruiting coactivators such as CREB-binding protein (CBP). This initiates the transcription of genes that are critical for multiple functions, including RUNX2, ITM2A, Bcl-2, BDNF, Mcl-1, and PGC-1α.^104^^,^^105^^,^^106^

The PKA-CREB signaling axis positively regulates ITM2A expression. Treatment with Forskolin, a PKA activator, significantly activates the ITM2A promoter and increases ITM2A expression. Furthermore, ITM2A interacts with v-ATPase, thereby inhibiting lysosomal function and subsequently disrupting autophagic flux.^12^

### Notch signaling pathway

The Notch signaling pathway is a highly conserved intercellular communication system across species. Its core mechanism relies on direct receptor-ligand interactions between neighboring cells, enabling precise regulation of cell fate decisions, including growth, differentiation, and stem cell self-renewal. Notch signaling is essential for both embryonic growth and adult tissue homeostasis, and its dysregulation is closely linked to a variety of diseases, particularly cancer. In mammals, four type I transmembrane Notch receptors (Notch1–4) undergo maturation in the Golgi apparatus, where constitutive S1 cleavage by a prolyl transpeptidase generates heterodimers that anchor to the cell surface, establishing the basis for subsequent signal initiation. Ligand-receptor binding occurs via direct cell-cell contact and serves as the activating switch of the pathway. This interaction induces a conformational change in the Notch receptor, revealing the S2 cleavage site within its extracellular domain. This site is subsequently cleaved by ADAM family metalloproteases (e.g., ADAM10 or TACE/ADAM17). This proteolytic event, often followed by *trans*-endocytosis of the Notch extracellular domain (NECD) into the ligand-presenting cell, results in the release of the extracellular segment. The remaining membrane-tethered fragment, known as NEXT (Notch extracellular truncation), undergoes S3 cleavage within the transmembrane domain by the γ-secretase complex. The resulting Notch intracellular domain (NICD) translocates into the nucleus, where it assembles with the DNA-binding protein CSL (also known as CBF1/RBP-Jκ) and coactivators from the Mastermind-like (MAML) family to form a transcriptional activation complex. This complex initiates the expression of downstream target genes, including key regulators such as Hes1/5, Hey1/2/L, Myc, SOX9, and NRARP.^107^^,^^108^^,^^109^

In cervical cancer, low ITM2A expression is associated with chemotherapy resistance and poor prognosis, and bioinformatic analyses suggest an association with the Notch signaling pathway. Overexpression of ITM2A significantly reduces both mRNA and protein levels of key Notch signaling components, including Notch1, Jagged1, Hes1, and c-Myc. Conversely, knockdown of ITM2A upregulates their expression. Rather than directly enhancing drug uptake, ITM2A suppresses the Notch pathway, resulting in decreased expression of DNA repair enzymes and anti-apoptotic proteins. This mechanism highlights ITM2A as a potential target for overcoming chemotherapy resistance in cervical cancer.^13^

### mTOR signaling pathway

mTOR is a serine/threonine protein kinase that belongs to the PIKK (phosphoinositide 3-kinase-related kinase) family. It responds to various upstream signals, including growth factors, energy status, and stress cues, and functions through two distinct complexes: mTORC1 and mTORC2. These complexes precisely regulate critical biological processes, including cell growth, metabolism, autophagy, and survival. Upstream regulators of mTORC1 include the PI3K-Akt pathway, Rag GTPases, AMPK, and HIF-1α, whereas mTORC2 is primarily induced by PI3K-Akt signaling. Downstream, mTORC1 phosphorylates 4E-BP1 (eukaryotic translation initiation factor 4E-binding protein 1) and S6K (ribosomal protein S6 kinase) to control protein synthesis. Under nutrient-rich conditions, mTORC1 also phosphorylates ULK1 (Unc-51 such as autophagy activating kinase 1) and ATG13, thereby suppressing autophagosome formation. Additionally, mTORC1 promotes lipogenesis through the activation of SREBP (sterol regulatory element-binding protein) and facilitates the synthesis of purines and pyrimidines by modulating relevant metabolic enzymes. mTORC2 exerts its effects primarily through the phosphorylation of Akt at S473, leading to full activation of Akt, inhibition of apoptosis, and enhanced cell survival. It also regulates actin cytoskeleton organization via phosphorylation of PKCα (protein kinase C alpha) and modulation of Rho GTPases, thereby influencing cell morphology and migration.^110^^,^^111^^,^^112^

In human breast cancer cells, ITM2A overexpression promotes autophagic flux in an mTOR-dependent manner, thereby inhibiting cancer cell proliferation. Specifically, ITM2A overexpression significantly suppresses mTOR activity, as indicated by decreased phosphorylation of T37/46 on 4E-BP1, which promotes autophagy. This effect occurs independently of the AMPK pathway, and autophagy induction depends on the phosphorylation state of ITM2A.^14^

## Participation in tissue development and cell maturation

### Cartilage and bone development

Bone develops via two primary processes: intramembranous osteogenesis and endochondral osteogenesis. The formation process involves several key steps: proliferation and differentiation of osteoprogenitor cells, development of osteoblasts, secretion of osteoid, embedding of osteoblasts within the osteoid, maturation into osteocytes, calcification of the bone matrix, and ultimately the formation of mature bone tissue. The major cell lineages involved in the formation of bone and cartilage include osteoblasts, osteocytes, and chondrocytes.^88^

ITM2A is expressed in multiple tissues, not only in structures of endochondral origin such as ribs, vertebrae, and long bones, but also in skeletal muscle, T cells, hair follicles, skin, and tongue tissue.^113^ Endochondral ossification, the primary mode of mammalian bone formation, progressively ossifies through cartilaginous templates and plays a crucial role in skeletal development, growth, and fracture repair.^20^^,^^114^ Studies have shown that ITM2A is expressed during the early stages of chondrocyte differentiation. In C3H10T1/2 mouse mesenchymal cells, *ITM2A* is preferentially expressed in the chondrogenic differentiation pathway, whereas its expression in the osteogenic pathway is relatively low. Similarly, in mouse MCT and ATDC5 chondrocyte cell lines, *ITM2A* expression is associated with early-stage chondrogenesis and negatively correlates with *collagen type X,* a marker of late-stage differentiation. Furthermore, overexpressing *ITM2A* in ATDC5 cells delayed *collagen type X* expression, suggesting that *ITM2A* overexpression may delay the onset of late-stage differentiation in these cells.^20^ *ITM2A* expression also varies between human stem cell populations. Levels are higher in adipose-derived stem cells (ASCs) than in bone marrow-derived mesenchymal stem cells (MSCs). During early chondrogenic induction, *ITM2A* expression is low in MSCs, whereas ASCs that fail to undergo chondrogenesis maintain higher *ITM2A* levels. This suggests that ITM2A may inhibit the initiation of chondrogenesis during the early stages of differentiation.^19^ During mouse embryonic development, ITM2A is expressed at an earlier stage than several classic endochondral ossification marker genes, including alkaline phosphatase (*ALP*), matrix metalloproteinase-13 (*MMP-13*), and osteocalcin (*OC*).^115^ In the growth plate, *ITM2A* is a suitable marker for non-hypertrophic chondrocytes in both the quiescent and proliferative zones, with higher expression in the quiescent zone and uneven distribution in the proliferative zone. Notably, *ITM2A* is not expressed in osteoblasts.^115^ In a mouse femoral fracture model, approximately 37% of the chondrocytes contributing to endochondral ossification originated from *ITM2A*^+^ cells.^11^ Endochondral ossification involves coordinated regulation of multiple secreted proteins (e.g., IHH, PTHrP, BMP, WNT, and FGF) and their receptors, along with transcription factors such as SOX9, RUNX2, and OSX.^114^ One study suggested that ITM2A may act as a physiological downstream target of the PTHrP signaling pathway, potentially mediating the PTHrP-induced phenotypic effects in chondrocytes.^115^ In summary, ITM2A plays a crucial role in the early stages of endochondral ossification. However, the precise mechanisms remain unclear, and studies in humans are limited. Further research is needed to elucidate the specific functions of ITM2A in endochondral bone formation.

Endochondral ossification generates bone through cartilage intermediates, whereas intramembranous ossification directly forms many craniofacial bones from mesenchymal condensations. ITM2A may participate, directly or indirectly, in the healing of cranial defects. Notably, adult cranial defects rarely heal spontaneously, while adolescents retain some capacity for ossification-based repair.^116^ Cranial healing in juvenile animals involves a complex regulatory network requiring the coordinated action of extracellular matrix proteins, growth factors, osteoblasts, and osteoprogenitor cells.^117^ Key structures in this repair process include cranial sutures, periosteum, dura mater, and periosteal tissues, with the dura mater, particularly in juveniles, playing a central role in cranial ossification.^117^^,^^118^ Compared to adult dura mater, juvenile dura mater exhibits significant upregulation of osteogenesis-related factors, differentiation markers, extracellular matrix components, and growth factors, indicating that immature dura mater provides a signaling environment essential for ossification.^119^ ITM2A itself serves as a key marker for osteogenic differentiation,^115^ exhibiting age-dependent expression patterns.^120^ Studies indicate that ITM2A expression in juvenile dura mater is approximately 17.1-fold higher than in adult samples,^119^ further suggesting its potential driving role in early ossification regulation. Moreover, ITM2A marks P-SSCs involved in skeletal development and regeneration. In a mouse femoral drilling injury model, *ITM2A*^+^ P-SSCs are the main source of osteoblasts during intramembranous ossification. *ITM2A*^+^ P-SSCs are also present in the human periosteum, and isolated *ITM2A*^+^ P-SSCs exhibit enhanced osteogenic potential *in vitro* and in mouse models.^11^

ITM2A expression and function exhibit age- and sex-dependent variations. Studies indicate that ITM2A escapes X chromosome inactivation (XCI) in most females, potentially contributing to height dimorphism.^121^^,^^122^ The differentiation capacity of bone marrow MSCs into bone, cartilage, and adipose tissue is related to age.^123^ The expression of ITM2A displays an age-dependent decline.^120^ In other words, aging is associated with reduced osteogenic and chondrogenic potential.^120^^,^^124^^,^^125^ Notably, elevated ITM2A levels may negatively regulate chondrogenic differentiation. Stephane et al. observed that the *ITM2A* expression profile dynamically changes in primary human bone marrow-derived MSCs, suggesting a role in inhibiting the initiation of chondrogenesis during early differentiation stages.^19^

### Myogenic differentiation and muscle regeneration

Three types of muscle tissue exist: skeletal muscle, cardiac muscle, and smooth muscle.^126^^,^^127^ Skeletal muscle accounts for about 40% of body weight and is among the largest organs in the human body. Composed of multinucleated muscle fibers, skeletal muscle plays a vital role in voluntary movement.^128^ Maintaining normal muscle function requires a balance between muscle damage and regeneration. It plays different roles at different stages and is responsible for the increase in cell number and cell size during development. Muscle function and size are maintained during adulthood through regeneration after muscle injury.^129^ Myogenic differentiation, a key process in muscle regeneration, occurs both during embryonic muscle formation and in adult muscle repair.^113^^,^^130^

Myogenic differentiation requires the induction of muscle-specific genes, such as *myosin*, and maintenance of cell cycle withdrawal.^102^^,^^131^^,^^132^ Endogenous ITM2A is necessary for timely induction of myogenic differentiation markers in murine myoblast C2C12 cells,^133^ and plays a crucial role *in vitro* differentiation.^102^^,^^113^ Its knockdown delays *in vitro* differentiation, while its overexpression promotes *in vitro* differentiation. Canonical Hh signaling is known to prevent myogenic differentiation, and selective autophagy plays a key role in the remodeling the mitochondrial network during this process.^102^^,^^134^^,^^135^ Cintli et al. found that during myogenic differentiation, basal autophagic flux was reduced and ITM2A expression was increased. ITM2A acts as a negative regulator of both canonical and non-canonical Hh signaling. It interacts with the Hh receptor PTCH1, and these two proteins independently inhibit autophagic flux. This confirms that ITM2A is crucial in myogenic differentiation.^102^ Additionally, IgLON5, a cell adhesion molecule in the immunoglobulin superfamily, is crucial for myogenesis.^136^ Its expression gradually increases during C2C12 differentiation, and knockdown of IgLON5 reduces ITM2A levels. Conversely, knockdown of ITM2A downregulates IgLON5, suggesting a reciprocal regulatory relationship between these two proteins during myogenic differentiation.^136^

Skeletal muscle satellite cells are muscle stem cells with strong proliferative and differentiative capabilities.^137^ In mouse models, ITM2A is essential for embryonic development and myogenesis, with its expression persisting in adult skeletal muscle satellite cells in both quiescent and activated states.^21^ PAX3, a key regulator of the nervous system, neural crest, and skeletal muscle development, directly targets *ITM2A in vivo*. *ITM2A* is expressed not only in adult muscle fibers but also in satellite cells responsible for regeneration, and its expression is influenced by myogenic determinants *Myf5* and *Mrf4*.^21^ miR-1, a skeletal muscle-specific microRNA, serves as a critical regulator of muscle development and homeostasis. In mouse models, miR-1 knockdown upregulates ITM2A expression and enhances the hypertrophic response, whereas miR-1 overexpression reduces ITM2A levels and abolishes hypertrophy, indicating that ITM2A is a potential target in miR-1-mediated regulation of muscle hypertrophy.^9^ ITM2A also participates in cardiac regeneration following ischemic injury in adult mammals. In mouse models, overexpression of the lncRNA CAREL impairs cardiac regeneration. lncRNA CAREL acts as a ceRNA for miR-296, and the overexpression of miR-296 promotes cardiomyocyte proliferation and cardiac regeneration. Furthermore, *ITM2A* and *Trp53inp1* are target genes of miR-296 that are positively regulated by lncRNA CAREL. Thus, the lncRNA CAREL-miR-296-ITM2A/Trp53inp1 axis is crucial for regulating cardiomyocyte proliferation and cardiac regeneration.^10^ Additionally, one study suggests that ITM2A may be associated with recurrent laryngeal neuropathy (RLN) in horses, a form of neuromuscular atrophy. It may contribute to RLN by regulating muscle physiology and growth.^138^

### Other functions

Beyond its roles in bone and muscle, ITM2A participates in a variety of physiological processes throughout the body. In tooth development, ITM2A has been demonstrated to be closely associated with enamel formation. As an interacting partner of enamel proteins, ITM2A may promote the formation of the enamel matrix by participating in the secretory pathway of ameloblasts. It is primarily expressed in the enamel epithelium, ameloblasts, and odontoblasts, indicating ITM2A is particularly important during the cellular differentiation stages of tooth development rather than during the initial formation of the tooth germ.^139^

ITM2A exerts important functions in multiple types of epithelial cells. Under normal physiological conditions, endothelial cells exhibit low selective permeability. Brain endothelial cells, in contrast to those in peripheral blood vessels, are tightly joined and possess numerous efflux systems, allowing only very small lipophilic compounds to cross the blood-brain barrier (BBB) while effectively blocking most macromolecules and polar substances. This unique barrier property presents a challenge for delivering biologics to the brain.^140^^,^^141^^,^^142^ Studies have shown that *ITM2A* is highly enriched in porcine brain microvascular endothelial cells (BMECs), with expression levels markedly higher than in whole brain tissue. In freshly isolated BMECs, *ITM2A* abundance is at least approximately 10-fold greater than in aortic endothelial cells, suggesting ITM2A may be BMEC-specifically expressed and involved in shaping their unique barrier phenotype.^143^ Further studies identified *ITM2A* as a transcript highly expressed in cerebral micro vessels, positioning it as a potential BBB-specific target.^144^ Analysis of mouse and human transcriptome datasets revealed that the *ITM2A* gene is uniquely expressed in brain endothelial cells, ranking among the most highly expressed genes in this cell type.^145^^,^^146^^,^^147^ Recent studies also indicate that ITM2A is functionally associated with the BBB. Antibodies specifically recognizing ITM2A show selective binding and uptake in ITM2A-overexpressing cells, highlighting its potential as a target for enhanced drug delivery to the human brain, although the validation of this approach remains complex.^141^ Additionally, ITM2A has been implicated in limbal epithelial stem cells and may contribute to the morphogenesis of limbal structures.^148^

*ITM2A* is a GATA-3 target gene that serves as a marker of T cell development. It is essential for the optimal development of OT-1 thymocytes and for the humoral response to T-helper cell-dependent antigens.^80^^,^^81^^,^^149^ Interestingly, ITM2A-deficient mice still exhibit normal development of T cells, B cells, and myeloid cells, likely due to functional compensation by other members within the ITM2 family.^80^ In addition, Magdalena et al. showed that ITM2A expression varies significantly during the culture process, suggesting a potential role in the growth of follicular granulosa cells.^150^

In summary, ITM2A is expressed during the early stages of bone formation and plays a crucial role in both endochondral and intramembranous ossification. However, most current research has focused on mouse models and phenotype-based studies, with limited investigation in humans. Future studies are needed to elucidate the specific mechanisms of ITM2A in endochondral and intramembranous ossification, providing a theoretical foundation for research on skeletal development and related disorders. It also plays a vital role in myogenic differentiation and muscle regeneration. Although some studies have demonstrated its potential mechanisms *in vivo*, existing research suffers from a lack of mechanistic depth and incomplete phenotypic analyses. Therefore, the specific functions and significance of ITM2A in this field require further clarification. Additionally, some studies have suggested that ITM2A may be involved in tooth development, BBB function, and T cell development. However, systematic analyses of its specific mechanisms in these areas are lacking as well. Overall, ITM2A likely performs important functions. These functions are related to the normal development and maturation of various tissues and cells within the body. Despite this, extensive further research is required due to the current lack of deep mechanistic research and incomplete phenotypic data.

## Involvement in disease initiation and development

### Cancers

**Figure 4:**
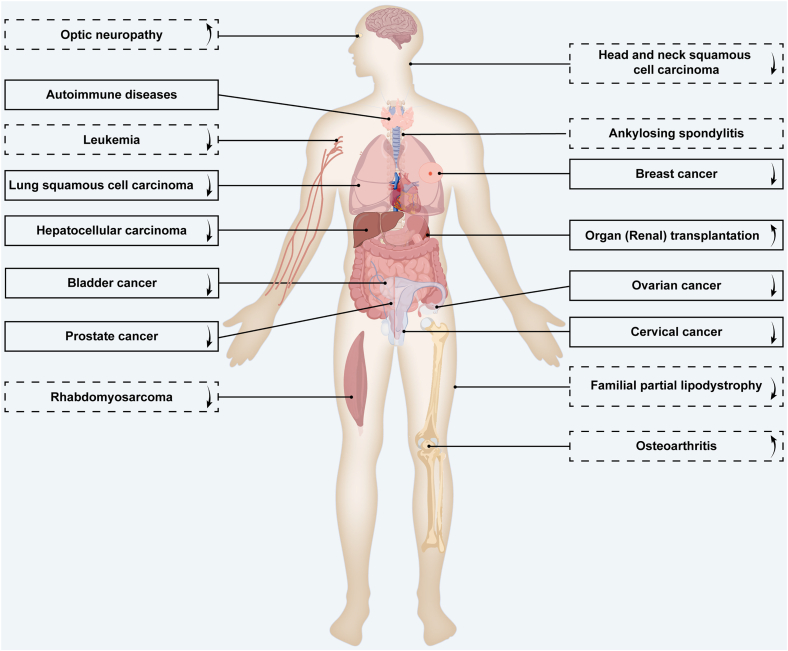
Differential expression and functions of ITM2A in human diseases ITM2A is upregulated in optic neuropathy in Ebola survivors, which may affect immune regulation; ITM2A is associated with autoimmune diseases, and its expression levels vary among different cell types; ITM2A may be associated with acute myeloid leukemia; ITM2A may be associated with rhabdomyosarcoma; ITM2A is associated with Lung squamous cell carcinoma, hepatocellular carcinoma, bladder cancer, prostate cancer, rhabdomyosarcoma, head and neck squamous cell carcinoma, breast cancer, ovarian cancer, and cervical cancer; ITM2A may be associated with ankylosing spondylitis and osteoarthritis; ITM2A may be associated with familial lipid metabolism disorders; ITM2A may associated with organ transplant rejection. In this context, solid boxes show that the association between ITM2A and the disease is confirmed, while dashed boxes show that the association requires further confirmation.

The incidence and mortality rates of cancer continue to rise rapidly worldwide, with breast, lung, pancreatic, and liver cancers among the most common types. Emerging evidence suggests that ITM2A may act as a tumor suppressor and could serve as a potential biomarker for prognosis and diagnosis for various cancers. Many studies indicate that ITM2A is involved in cancer progression and is usually downregulated in tumor tissues (Figure 4). While research on ITM2A in cancer is limited and lacks depth, existing studies suggest it plays a critical role, particularly within the tumor immune microenvironment (TIME). Furthermore, a few studies have reported that ITM2A-targeted small-molecule drugs can effectively inhibit cancer progression. Consequently, in-depth investigation of ITM2A is clinically significant, as it may inform personalized therapeutic strategies and improve prognosis for patients with cancer.

### Breast cancer

ITM2A shows considerable potential in influencing disease progression, prognostic assessment, and molecular therapy for breast cancer. Studies indicate its expression is markedly downregulated in human breast cancer tissues and cell lines, with low expression closely associated with clinical-pathological parameters, including reduced overall survival, progesterone receptor status, TNM staging, and unfavorable tumor grading.^14^^,^^151^ Conversely, patients with high ITM2A expression display longer overall survival and recurrence-free survival.^14^^,^^151^ Triple-negative breast cancer (TNBC), a highly aggressive subtype of breast cancer,^152^^,^^153^ also exhibits significantly reduced ITM2A levels. Patients with low ITM2A expression in TNBC have poorer overall survival, recurrence-free survival, and distant metastasis-free survival.^152^^,^^153^ Functional studies further confirm that ITM2A overexpression significantly inhibits proliferation and promotes apoptosis of breast cancer cells, and reduces their invasive and migratory capabilities in both *in vitro* and *in vivo* models.^14^^,^^151^

ITM2A plays a critical role in suppressing tumor proliferation by modulating autophagy. Autophagy is a conserved physiological process in which damaged proteins or organelles in eukaryotic cells are degraded via lysosomal pathways, with the resulting products recycled for cellular use.^154^ This process is essential for maintaining cell homeostasis, growth, self-renewal, aging, immunity, tumorigenesis, and the pathogenesis of neurodegenerative diseases. Autophagy can be categorized into three types based on the enclosed material and transport mechanism: macro autophagy, micro autophagy, and chaperone-mediated autophagy, with macro autophagy generally referred to as autophagy.^155^ Key signaling pathways regulating autophagy include AMPK-mTOR, Class I PI3K/Akt/mTOR, and MAPK/ERK signaling pathways.^156^^,^^157^^,^^158^^,^^159^ ITM2A expression is significantly reduced in human breast cancer cells and tissues, and database analyses indicate that low ITM2A levels correlate with poor clinical and pathological outcomes.^14^ AMPK and mTOR serve as core regulators of autophagy.^157^ ITM2A has limited effects on AMPK activity, but its overexpression significantly inhibits mTOR activation. As mTOR is a key driver of cell proliferation, the suppression of mTOR by ITM2A is relieved in breast cancers with downregulated ITM2A expression, thereby promoting tumor growth. Additionally, serine/threonine kinase HUNK phosphorylates ITM2A at T35, a modification critical for autophagy induction.^14^

Beyond its role in autophagy, ITM2A also plays a critical role in tumor progression by modulating TIME. TIME is a complex and dynamic ecosystem made up of tumor cells, immune cells, the extracellular matrix, cytokines, and metabolic byproducts. It is closely associated with tumor initiation, progression, and treatment response.^160^ In breast cancer, RNA-seq of cells overexpressing ITM2A or cells lacking ITM2A revealed a significant enrichment of immune-related responses. ITM2A was found to induce the upregulation of immune checkpoint molecules, including PD-L1, PD-L2, and B7-H3. Additionally, analysis of the TIMER database revealed a positive correlation between ITM2A expression and tumor-infiltrating lymphocytes (TILs), including B cells, CD8^+^ T cells, CD4^+^ T cells, macrophages, neutrophils, and dendritic cells.^151^ In TNBC, a combined multi-omics analysis revealed that ITM2A coordinates antitumor immune responses by modulating copper ion metabolism reprogramming and the immune checkpoint network. Specifically, the expression levels of ITM2A correlate positively with those of more than 60 classic immune checkpoint molecules, including PD-L1, CTLA-4, and LAG-3. High ITM2A expression is associated with increased immune cell infiltration, including CD8^+^ T cells, CD4^+^ T cells, B cells, and macrophages, and enhanced sensitivity to immunotherapies such as anti-PD-L1 therapy and CAR-T cell therapy.^153^

Currently, reports on ITM2A-targeted therapies in breast cancer are limited. Etoposide, a cell cycle-specific antineoplastic agent, is commonly used to treat TNBC. Studies have shown that etoposide binds strongly to ITM2A, and overexpression of ITM2A significantly reduces the IC50 (half-maximal inhibitory concentration) of etoposide in HCC1806 cells, indicating enhanced drug sensitivity.^153^

In summary, ITM2A has an inhibitory effect on breast cancer, and its low expression is associated with a more malignant phenotype. ITM2A influences breast cancer development, progression, and prognosis by regulating autophagy and modulating the TIME. TNBC, a subtype of breast cancer with ineffective therapeutic targets, high chemotherapy resistance, and poor prognosis, is particularly influenced by ITM2A, where higher expression exerts protective effects. Additionally, ITM2A binds to etoposide, enhancing the sensitivity of breast cancer cells to the drug. These findings suggest that ITM2A holds potential as a biomarker for evaluation, diagnosis, prognosis, and therapy in breast cancer. However, despite evidence of its anticancer effects *in vitro* and *in vivo*, comprehensive data from cellular and animal models remain limited. Future studies should systematically investigate the specific mechanisms of ITM2A across different molecular subtypes of breast cancer to facilitate the development of ITM2A-based personalized therapeutic strategies.

### Ovarian cancer

In ovarian cancer, ITM2A expression is significantly downregulated in both ovarian cancer tissues and cell lines. Loss of ITM2A expression was observed in 45.6% of invasive serous ovarian cancer tissue samples. This loss of expression is significantly positively correlated with higher FIGO stage, type II tumors, suboptimal cytoreductive surgery status, disease recurrence, and chemotherapy resistance. These findings suggest that ITM2A loss may serve as a marker for predicting patient response to standard treatment.^15^ *In vitro* studies demonstrate that ITM2A exerts antitumor effects and enhances chemosensitivity. Higher ITM2A expression is associated with slower ovarian cancer cell proliferation and increased sensitivity to paclitaxel or carboplatin. Mechanistically, ITM2A induces G2/M phase arrest by downregulating key cell cycle regulators, including cdc2, *p*-cdc2 (T161), cdc25c, and cyclin B1 ^15^. In mouse models, overexpression of ITM2A significantly inhibited tumor growth and reduced the protein levels of Cdc2, P-Cdc2, Cdc25C, and cyclin B1. When combined with paclitaxel treatment, mice with high ITM2A expression exhibited notable reductions in tumor weight.^15^ Overall, high levels of ITM2A are associated with enhanced antitumor activity and greater chemosensitivity in ovarian cancer.

### Cervical cancer and uterine sarcoma

Cervical cancer is a major malignancy threatening women’s health, with a high mortality rate.^161^^,^^162^ *ITM2A*, a gene recently identified as associated with cervical cancer, plays a significant role in tumor development and progression. Studies indicate that low ITM2A expression correlates with poor prognosis in patients with cervical adenocarcinoma, suggesting it as a potential biomarker for cervical cancer classification and prognostic assessment.^13^^,^^163^ Furthermore, ITM2A expression closely correlates with the histological subtype of cervical cancer: Low expression is more frequent in adenocarcinoma, whereas high expression is predominantly observed in squamous cell carcinoma. Given that adenocarcinoma generally carries a poorer prognosis than squamous cell carcinoma, ITM2A expression analysis provides valuable guidance for individualized treatment strategies and prognosis.^163^ Cervical squamous cell carcinoma (CESC) is the most common histological subtype. Huang yang et al. developed a linear risk model for CESC incorporating five candidate genes (*DSG2*, *ITM2A*, *CENPM*, *RIBC2*, and *MEIS2*), demonstrating significant prognostic predictive capability.^164^ Notably, cisplatin treatment significantly reduced ITM2A expression in recurrent cervical cancer samples, and similar reductions were observed in cisplatin-resistant cervical cancer cell lines. Functional experiments revealed that ITM2A overexpression significantly decreased the IC50 and colony formation in the cisplatin-resistant SIHA cell line, whereas ITM2A knockdown produced the opposite effect.^13^

Beyond cervical cancer, ITM2A exhibits differential expression in other tumors. Endometrial stromal sarcoma (ESS), a rare type of uterine sarcoma, shows overexpression of ITM2A. ESS, together with leiomyosarcoma (LMS), constitutes the two most common forms of uterine sarcoma.^165^

Overall, ITM2A is expressed at low levels in cervical cancer but is highly expressed in ESS. Increased ITM2A expression enhances the sensitivity of cervical cancer cells to cisplatin. However, research on ITM2A in both cervical cancer and ESS remains in its early stages. Future studies are needed to elucidate the specific mechanisms of ITM2A in these cancers, providing a theoretical foundation for the development and application of molecular-targeted therapies.

### Hepatocellular carcinoma

Liver cancer, particularly hepatocellular carcinoma (HCC), is among the most common and lethal malignancies worldwide. In patients with HCC, ITM2A expression levels are closely associated with prognosis, demonstrating ITM2A as a potential prognostic biomarker. One study constructed a prognostic model incorporating eight genes, including H2AFX, SQSTM1, ITM2A, PFKP, TPD52L1, ACSL4, STRN3 and CPEB3, to predict overall survival in patients with HCC, which may help assess individual mortality risk.^166^ Ferroptosis, a form of regulated cell death that inhibits tumor proliferation and modulates the TIME, also involves ITM2A. A separate model based on ten ferroptosis-related genes, including ITM2A, identified its potential role in HCC progression.^167^ ITM2A is recognized as a key T cell proliferation-related gene (TRG) and serves as a candidate biomarker for colorectal cancer (CRC).^168^ Analysis of TCGA-LIHC data further indicated that higher ITM2A expression correlates with better prognosis, increased T cell infiltration, and a lower recurrence rate in HCC. Single-cell RNA sequencing (scRNA-seq) revealed that ITM2A is primarily expressed in T cell and NK-cell subtypes, underscoring its potential role in antitumor immunity.^169^

TCR/IL-2 signaling plays a critical role in T cell activation, expansion, and homeostasis. Studies using gene knockout and conditional knockout mouse models revealed that ITM2A deficiency impairs TCR signaling in TILs within the tumor microenvironment (TME). ITM2A enhances TCR signaling by promoting CD3ζ-induced ZAP70 phosphorylation. Conversely, tumor-infiltrating macrophages can induce CD4^+^ T cell exhaustion by disrupting the ITM2A-ZAP70 interaction via MSR1-ITM2A. Through screening of small-molecule libraries, zeaxanthin (ZEA) was identified as a potent inhibitor of the MSR1-ITM2A interaction both *in vitro* and *in vivo*. Systemic administration of ZEA effectively suppresses tumor progression, reduces CD4^+^ T cell exhaustion, and limits the infiltration of immunosuppressive myeloid cells. Based on these findings, a novel antibody-drug conjugate (ADC), ZEA-αCD4, was developed. This ADC inhibits tumor growth and enhances antitumor immune responses within the TME. Furthermore, combining the ADC with αPD-L1 and apatinib (a standard HCC treatment) or with αPD-L1/αCTLA plus apatinib further enhances antitumor efficacy.^169^

In summary, ITM2A functions as a tumor suppressor in HCC and plays a role in the TIME. Studies have shown that the small-molecule drug ZEA exhibits significant antitumor activity against HCC, with efficacy independent of conventional αPD-L1 immunotherapy. ZEA may therefore be effective in patients resistant to existing PD-1/PD-L1 therapies and could be used in combination with PD-L1 inhibitors to enhance therapeutic outcomes. Furthermore, ITM2A may serve as a prognostic marker in HCC. Investigating its mechanistic roles could have important implications for clinical diagnosis, personalized therapy, and prognostic assessment in patients with HCC.

### Other cancers

HNSCC (Head and neck squamous cell carcinoma) is a highly malignant tumor closely related to the TIME. Bioinformatic analyses have revealed that ITM2A is aberrantly expressed across various cancers and demonstrates significant correlations with TIME components, including immune infiltrating cells, immune checkpoints, and responses to immunotherapy. scRNA-seq further detected ITM2A expression across multiple immune cell subsets within the TIME, suggesting a potential role in modulating immune responses in HNSCC.^170^ Moreover, ITM2A is downregulated in tumor and dysplastic tissues, likely due to hypermethylation at multiple methylation sites, and may serve as a potential indicator for clinical staging and prognostic assessment in HNSCC.^171^

LUSC is a highly aggressive malignancy with poor prognosis. Studies have shown that circITFG2 overexpression inhibits LUSC cell proliferation, migration, and invasion *in vitro*, and significantly suppresses tumor growth *in vivo*. RNA-seq analyses indicate that ITM2A is a critical downstream target of circITFG2, enriched in immune-related processes. In mouse models, circITFG2 overexpression can reverse the oncogenic effects caused by ITM2A knockdown. Furthermore, ITM2A expression negatively correlates with PD-L1 levels. Mechanistically, circITFG2 upregulates ITM2A by sponging miR-526b-5p, which activates autophagy and promotes PD-L1 degradation, ultimately enhancing antitumor immune responses. In summary, circITFG2 promotes ITM2A-mediated autophagy and PD-L1 degradation, thereby strengthening immune-mediated tumor suppression.^78^

In bladder cancer, low ITM2A expression is significantly associated with higher tumor grade, later AJCC stage, and poorer overall survival. Functional studies show that ITM2A inhibits proliferation, migration, and invasion of bladder cancer cells by suppressing IL-6-induced STAT3 activation. In mouse models, overexpression of ITM2A suppressed tumor growth *in vivo*.^85^ Additional studies demonstrated that ITM2A promotes apoptosis, upregulates the epithelial marker E-cadherin, and downregulates the mesenchymal marker N-cadherin, suggesting a role in modulating epithelial-mesenchymal transition (EMT).^172^ EMT is regulated by multiple signaling pathways, including TGF-β, Wnt, and Notch.^173^ Therefore, ITM2A may modulate EMT by influencing one or more of these pathways, although the specific upstream mechanisms remain to be experimentally validated.

Analysis of the TCGA datasets revealed that ITM2A is downregulated in prostate cancer tissues. Functional studies further demonstrated that overexpression of ITM2A significantly inhibited tumor growth in mouse models, highlighting its potential tumor-suppressive role.^84^

In acute myeloid leukemia (AML), high ITM2A expression is associated with improved overall survival.^174^ Furthermore, as a downstream target of GATA3, ITM2A is also implicated in the progression of B-cell acute lymphoblastic leukemia (B-ALL).^175^ Analysis of CML patient samples revealed that ITM2A expression is low in tyrosine kinase inhibitor (TKI)-resistant patients and high in TKI-sensitive patients and healthy controls. *In vitro*, ITM2A levels were significantly lower in the imatinib-resistant CML cell line K562R compared with the imatinib-sensitive K562 cells. Additionally, overexpressing ITM2A increased the sensitivity of K562R cells to imatinib and stopped the cell cycle of K562R cells in the G2 phase. Mechanistically, this effect is likely mediated through the suppression of ERK phosphorylation, indicating that ITM2A may modulate drug resistance via the ERK signaling pathway.^83^

PAX3-FKHR is a key oncogenic driver of alveolar rhabdomyosarcoma (ARMS).^176^ Through screening of mouse and human PAX3/PAX3-FKHR libraries, *ITM2A* was identified as a potential downstream target. Its expression correlates with PAX3 levels in mouse embryos and with PAX3-FKHR levels in rhabdomyosarcoma cell lines, suggesting that *ITM2A* may be a PAX3-FKHR target gene involved in tumorigenesis.^81^

### Bone disorders

ITM2A plays a critical role in skeletal development and the maintenance of bone homeostasis, exhibiting specific expression patterns in various skeletal disorders (Figure 4), thereby demonstrating its potential as a target for bone-related diseases. Giordano et al. reported a case involving a 5.8 Mb mesenchymal deletion on the Xq21.1 chromosomal region encompassing the *ITM2A* gene locus. This deletion severely disrupted normal cartilage development and was likely associated with the patient’s severe short stature phenotype.^177^

A study using scRNA-seq analysis of *Prrx1-Cre*; *R26-Ai9* mice found that *ITM2A* is specifically enriched in *p*-SSCs, serving as a reliable marker for this population. In a mouse fracture model, *ITM2A*^+^ lineage cells were identified as the primary source of chondrocytes and osteoblasts during fracture healing. Furthermore, either *BMP2* deficiency or ablation of *ITM2A*^+^ P-SSCs resulted in impaired fracture repair, highlighting the critical role of ITM2A in skeletal regeneration.^11^

OA is a common degenerative joint disease characterized by the progressive destruction of articular cartilage.^178^^,^^179^ In a study analyzing articular cartilage samples from 25 patients with OA and 10 healthy donors, *ITM2A* expression was found to be significantly elevated in OA cartilage compared with healthy tissue.^179^

Ankylosing spondylitis (AS) is an immune-mediated disease characterized by chronic inflammation of the spine and sacroiliac joints, with strong genetic susceptibility, particularly HLA genes, and influence from multiple environmental factors.^180^^,^^181^^,^^182^ Studies investigating ITM2A expression in AS have produced conflicting results. One analysis of samples from 31 patients with AS and 39 controls reported significantly higher ITM2A expression in patients with AS.^183^ Conversely, a differential gene expression study involving 16 patients with AS and 16 controls found lower ITM2A expression in patients with AS.^184^ To address these discrepancies, a follow-up study of 14 patients with AS and 14 healthy controls examined macrophages and found that both basal and inflammation-induced ITM2A expression were significantly downregulated in patients with AS.^181^ Despite these conflicting expression trends, it is clear that ITM2A dysregulation occurs in AS. ITM2A may contribute to immune regulation and the metabolic balance of bone and cartilage, potentially through the modulation of autophagy pathways.

In summary, case reports and expression analyses indicate that ITM2A plays a significant role in bone-related diseases. Its expression patterns in OA and AS further suggest that ITM2A may participate in immune regulation within these conditions. However, current research is limited and lacks depth, and the specific mechanisms by which ITM2A contributes to these diseases remain unclear. Future studies should aim to elucidate the molecular pathways through which ITM2A regulates immunity in bone-related inflammatory disorders and explore its clinical applications, potentially offering new strategies for the treatment of OA and AS.

### Autoimmune thyroid diseases

Graves' disease (GD) and Hashimoto’s thyroiditis (HT) are the two primary clinical subtypes of autoimmune thyroid disease (AITD), with their pathogenesis influenced by both genetic predisposition and environmental factors.^185^^,^^186^ Gender differences are particularly pronounced, with about 80% of patients with AITD being female.^187^ Skewed XCI is considered a key mechanism contributing to the elevated risk of AITD in women.^188^^,^^189^^,^^190^ Since many genes on sex chromosomes exhibit sex-specific expression, females are more prone to autoimmune diseases than males.^191^ In a study analyzing 4316 patients with GD and 4374 gender-matched controls, PBMCs were collected, and genomic DNA was examined. The results suggested that rs3827440, or a closely linked SNP, is likely the most significant variant in the Xq21.1 region contributing to GD susceptibility. Further analysis revealed that ITM2A expression in monocytes correlates with rs3827440, indicating that this variant and its associated SNPs may increase GD risk by regulating ITM2A expression. Environmental stimuli, such as bacterial or viral infections, may further modulate this effect. Upon the stimulation of monocytes with LPS or IFN-γ, the correlation between expression-affecting SNPs (eSNPs) and ITM2A expression was significantly enhanced. In summary, *ITM2A* is a GD susceptibility gene (Figure 4), and its expression is regulated by rs3827440. Environmental factors, such as infection, may also induce GD through this mechanism.^8^

### Other diseases

ITM2A plays a crucial role in the progression of multiple diseases. Beyond its involvement in tumorigenesis and bone and thyroid disorders, ITM2A also participates in a range of other pathological processes (Figure 4).

Denny-Dunn familial partial lipodystrophy type 2 (FPLD2) is an autosomal dominant laminopathy characterized primarily by impaired lipid metabolism and storage, caused by pathogenic mutations in LMNA.^192^^,^^193^ In 3T3-L1 preadipocytes overexpressing LMNA (WT or R482W), adipogenesis was significantly reduced. Following the induction of differentiation in these cells, RNA-seq and quantitative real-time PCR (qPCR) analyses revealed significantly increased ITM2A expression. Conversely, ITM2A levels were markedly reduced in LMNA-knockout mouse embryonic fibroblasts (MEFs). During 3T3-L1 differentiation, ITM2A expression decreased initially and returned to pre-induction levels by day 4. Functional studies demonstrated that ITM2A overexpression inhibited adipogenesis, whereas ITM2A knockdown enhanced adipogenesis and rescued the LMNA-mediated suppression. Additionally, ITM2A knockdown led to the accumulation of LMNA protein, indicating a regulatory feedback loop. Together, these findings indicate that ITM2A is a key downstream mediator of LMNA in adipocyte differentiation: LMNA upregulates ITM2A to suppress adipogenesis. These results suggest a potential role for ITM2A in metabolic diseases.^193^

Acute rejection is a common complication in organ transplantation, and multiple studies have highlighted the role of *ITM2A* in clinical allograft rejection.^16^^,^^194^^,^^195^^,^^196^ Although kidney transplantation has markedly improved short-term graft survival, acute rejection remains a major risk factor for transplant failure. Interestingly, *ITM2A* is specifically enriched in the urine of patients with T cell-mediated rejection (TCMR) and antibody-mediated rejection (AMR), while no corresponding enrichment is observed in kidney biopsy tissues.^16^ In lung transplant patients, *ITM2A* expression was found to be upregulated in bronchial biopsies from individuals experiencing acute lung rejection.^194^

A case-control study conducted in the Democratic Republic of the Congo from 2018 to 2020 found that Ebola virus disease (EVD) survivors with optic neuropathy exhibited significantly higher ITM2A expression compared to individuals without optic neuropathy. Further analysis suggested that aberrant ITM2A expression may contribute to the development and progression of optic neuropathy, indicating that ITM2A could play a role in the pathological processes underlying post-EVD optic neuropathy.^197^

Both chronic inflammatory demyelinating Polyneuropathy (CIDP) and Guillain-Barré Syndrome (GBS) are rare neurological disorders. In a proteomic analysis of serum samples from 11 healthy controls, 21 patients with GBS, and 19 patients with CIDP, ITM2A expression was found to be significantly elevated in patients with CIDP compared to patients with GBS, in whom no significant changes were observed.^198^

## Conclusion

ITM2A is widely expressed in multiple tissues, and its expression is precisely regulated by multidimensional factors. It plays a vital part in the maintenance of normal growth and development processes, such as bone formation, cartilage development and muscle differentiation. Additionally, ITM2A participates in various disease processes through distinct molecular mechanisms. Its expression is context-dependent: It is generally downregulated in cancers but upregulated in inflammatory conditions such as OA, suggesting that ITM2A may exert differential roles across pathological contexts.

While current research has partially elucidated ITM2A’s functions and outlined its associated regulatory networks and signaling pathways, a comprehensive understanding of its systemic mechanisms remains incomplete. This paper systematically elucidates the biological functions of the BRICHOS superfamily, whose members primarily exert molecular chaperone activities through the conserved BRICHOS domain, collectively forming a protein family that plays a central role in maintaining protein homeostasis. As a key member of this family, ITM2A’s *in vivo* regulatory network and its roles in growth, development, and disease processes have been preliminarily characterized. However, compared to other family members, such as ITM2B, ITM2C, and ProSP-C, the molecular chaperone mechanisms of ITM2A remain largely unexplored, highlighting a critical avenue for investigating its novel biological functions.

Research on the role of ITM2A in disease is still in its early stages, but emerging evidence suggests that it may play a critical regulatory role in various pathological processes. Systematic reviews have shown a correlation between ITM2A expression or activity and disease progression. This indicates ITM2A may serve as a candidate biomarker for diagnostic and prognostic purposes. Moreover, ITM2A may serve as a promising therapeutic target. Future studies should focus on elucidating the specific molecular mechanisms by which ITM2A contributes to disease development and assessing the therapeutic potential of targeting this molecule. Such investigations could facilitate the development of personalized treatment strategies based on ITM2A subtypes, ultimately improving intervention approaches for related diseases. Such investigations could facilitate the development of personalized treatment strategies based on ITM2A subtypes, ultimately improving intervention approaches for related diseases.

## Data and code availability

The submitted article is a review and does not have any associated data files. Therefore, data sharing is not applicable in this context.

## Acknowledgments

We also thank Hubei University of Technology and the University of Alberta for financial support for this research.

Funding: This work was supported by the 10.13039/501100001809National Natural Science Foundation of China (82273970 to J.F.T., 82370715 to X.Z.C., 32270768 to C.F.Z.), the 10.13039/501100000038Natural Sciences and Engineering Research Council of Canada (10.13039/501100000038NSERC), the 10.13039/501100000191Kidney Foundation of Canada (RGPIN-2019-05953 and 2020KHRG-673101 to X.Z.C.), and the 10.13039/501100012166National Key R&D Program of China (2023YFC2507900 to J.F.T.), the Innovation Group Project of Hubei Province (2023AFA026 to J.F.T.), the Key Cultivation Project of Hubei Province for Science and Technology (2024DJA037 to J.F.T.), and the National Natural Science Foundation of Hubei (2025AFA085 to C.F.Z.).

## Author contributions

Conceptualization, visualization, and methodology: X.L.D. and Y.F.W. writing – original draft: X.L.D., Y.F.W., Y.L., and K.L.W. writing – review and editing: W.Y.Q., D.W.A., M.M., J.F.T., C.F.Z., and X.Z.C. conceptualization, supervision, funding acquisition, and project administration: J.F.T., C.F.Z., and X.Z.C.

## Declaration of interests

The authors declare no competing interests.

## Declaration of generative AI and AI-assisted technologies in the writing process

During the preparation of this work, the authors did not use any AI-assisted technologies.

## References

[1] Sánchez-Pulido L., Devos D., Valencia A. (2002). BRICHOS: a conserved domain in proteins associated with dementia, respiratory distress and cancer. Trends Biochem. Sci..

[2] Hedlund J., Johansson J., Persson B. (2009). BRICHOS - a superfamily of multidomain proteins with diverse functions. BMC Res. Notes.

[3] Sáenz A., Presto J., Lara P., Akinyi-Oloo L., García-Fojeda B., Nilsson I., Johansson J., Casals C. (2015). Folding and Intramembraneous BRICHOS Binding of the Prosurfactant Protein C Transmembrane Segment. J. Biol. Chem..

[4] Leppert A., Poska H., Landreh M., Abelein A., Chen G., Johansson J. (2023). A new kid in the folding funnel: Molecular chaperone activities of the BRICHOS domain. Protein Sci..

[5] Jia Q.H., Cao Y.Z., Xing Y.X., Ma C.L., Guan H.B., Wang Q., Kang X.T., Tian Y.D., Li Z.J., Liu X.J., Li H. (2024). Biological Characteristics of Chicken (*Gallus gallus) ITM2A* Gene and Its Role in Myoblast Proliferation and Differentiation. J. Agricult. Biotechnol..

[6] Deleersnijder W., Hong G., Cortvrindt R., Poirier C., Tylzanowski P., Pittois K., Van Marck E., Merregaert J. (1996). Isolation of markers for chondro-osteogenic differentiation using cDNA library subtraction. Molecular cloning and characterization of a gene belonging to a novel multigene family of integral membrane proteins. J. Biol. Chem..

[7] Wu S.S., Chen J., Wang Y.C., Ma Y.X., Wei S.Y., Liu H.Y., Dong Y.Z., Wang B.Y., Cui Y.D., Ma J.Z. (2023). Screening of differentially expressed genes in mouse macrophages regulated by Mn2+ based on mRNA-seq data. Academic Journal of Naval Medical University.

[8] Ye X.P., Yuan F.F., Zhang L.L., Ma Y.R., Zhang M.M., Liu W., Sun F., Wu J., Lu M., Xue L.Q. (2017). ITM2A Expands Evidence for Genetic and Environmental Interaction in Graves Disease Pathogenesis. J. Clin. Endocrinol. Metab..

[9] Fei S., Rule B.D., Godwin J.S., Mobley C.B., Roberts M.D., von Walden F., Vechetti I.J. (2025). miRNA-1 regulation is necessary for mechanical overload-induced muscle hypertrophy in male mice. Phys. Rep..

[10] Cai B., Ma W., Ding F., Zhang L., Huang Q., Wang X., Hua B., Xu J., Li J., Bi C. (2018). The Long Noncoding RNA CAREL Controls Cardiac Regeneration. J. Am. Coll. Cardiol..

[11] Xing W., Feng H., Jiang B., Gao B., Liu J., Xie Z., Zhang Y., Hu X., Sun J., Greenblatt M.B. (2024). Itm2a expression marks periosteal skeletal stem cells that contribute to bone fracture healing. J. Clin. Investig..

[12] Namkoong S., Lee K.I., Lee J.I., Park R., Lee E.J., Jang I.S., Park J. (2015). The integral membrane protein ITM2A, a transcriptional target of PKA-CREB, regulates autophagic flux via interaction with the vacuolar ATPase. Autophagy.

[13] Li Y., Wang J., Gao C., Hu Q., Mao X. (2021). Integral membrane protein 2A enhances sensitivity to chemotherapy via notch signaling pathway in cervical cancer. Bioengineered.

[14] Zhou C., Wang M., Yang J., Xiong H., Wang Y., Tang J. (2019). Integral membrane protein 2A inhibits cell growth in human breast cancer via enhancing autophagy induction. Cell Commun. Signal..

[15] Nguyen T.M.H., Shin I.W., Lee T.J., Park J., Kim J.H., Park M.S., Lee E.J. (2016). Loss of ITM2A, a novel tumor suppressor of ovarian cancer through G2/M cell cycle arrest, is a poor prognostic factor of epithelial ovarian cancer. Gynecol. Oncol..

[16] Dooley B.J., Verma A., Ding R., Yang H., Muthukumar T., Lubetzky M., Shankaranarayanan D., Elemento O., Suthanthiran M. (2020). Urinary Cell Transcriptome Profiling and Identification of ITM2A, SLAMF6, and IKZF3 as Biomarkers of Acute Rejection in Human Kidney Allografts. Transplant. Direct.

[17] Willander H., Hermansson E., Johansson J., Presto J. (2011). BRICHOS domain associated with lung fibrosis, dementia and cancer--a chaperone that prevents amyloid fibril formation?. FEBS J..

[18] Cho W.K., Baek I.-C., Kim S.E., Kim M., Kim T.G., Suh B.K. (2023). Association of ITM2A rs1751094 polymorphism on X chromosome in Korean pediatric patients with autoimmune thyroid disease. Immun. Inflamm. Dis..

[19] Boeuf S., Börger M., Hennig T., Winter A., Kasten P., Richter W. (2009). Enhanced ITM2A expression inhibits chondrogenic differentiation of mesenchymal stem cells. Differentiation.

[20] Van den Plas D., Merregaert J. (2004). In vitro studies on Itm2a reveal its involvement in early stages of the chondrogenic differentiation pathway. Biol. Cell.

[21] Lagha M., Mayeuf-Louchart A., Chang T., Montarras D., Rocancourt D., Zalc A., Kormish J., Zaret K.S., Buckingham M.E., Relaix F. (2013). Itm2a is a Pax3 target gene, expressed at sites of skeletal muscle formation in vivo. PLoS One.

[22] Mitsui S., Osako Y., Yuri K. (2014). Mental retardation-related protease, motopsin (prss12), binds to the BRICHOS domain of the integral membrane protein 2a. Cell Biol. Int..

[23] Haqqani A.S., Bélanger K., Stanimirovic D.B. (2024). Receptor-mediated transcytosis for brain delivery of therapeutics: receptor classes and criteria. Front. Drug Deliv..

[24] Yasukawa T., Tsutsui A., Tomomori-Sato C., Sato S., Saraf A., Washburn M.P., Florens L., Terada T., Shimizu K., Conaway R.C. (2020). NRBP1-Containing CRL2/CRL4A Regulates Amyloid beta Production by Targeting BRI2 and BRI3 for Degradation. Cell Rep..

[25] Peng S., Fitzen M., Jörnvall H., Johansson J. (2010). The extracellular domain of Bri2 (ITM2B) binds the ABri peptide (1-23) and amyloid beta-peptide (Abeta1-40): Implications for Bri2 effects on processing of amyloid precursor protein and Abeta aggregation. Biochem. Biophys. Res. Commun..

[26] Rengaraj D., Gao F., Liang X.H., Yang Z.M. (2007). Expression and regulation of type II integral membrane protein family members in mouse male reproductive tissues. Endocrine.

[27] Latil A., Chêne L., Mangin P., Fournier G., Berthon P., Cussenot O. (2003). Extensive analysis of the 13q14 region in human prostate tumors: DNA analysis and quantitative expression of genes lying in the interval of deletion. Prostate.

[28] Baron B.W., Baron R.M., Baron J.M. (2015). The ITM2B (BRI2) gene is a target of BCL6 repression: Implications for lymphomas and neurodegenerative diseases. Biochim. Biophys. Acta.

[29] Dolfe L., Tambaro S., Tigro H., Del Campo M., Hoozemans J.J.M., Wiehager B., Graff C., Winblad B., Ankarcrona M., Kaldmäe M. (2018). The Bri2 and Bri3 BRICHOS Domains Interact Differently with Abeta(42) and Alzheimer Amyloid Plaques. J. Alzheimers Dis. Rep..

[30] Pittois K., Deleersnijder W., Merregaert J. (1998). cDNA sequence analysis, chromosomal assignment and expression pattern of the gene coding for integral membrane protein 2B. Gene.

[31] Vidal R., Calero M., Révész T., Plant G., Ghiso J., Frangione B. (2001). Sequence, genomic structure and tissue expression of Human BRI3, a member of the BRI gene family. Gene.

[32] Poska H., Leppert A., Tigro H., Zhong X., Kaldmäe M., Nilsson H.E., Hebert H., Chen G., Johansson J. (2020). Recombinant Bri3 BRICHOS domain is a molecular chaperone with effect against amyloid formation and non-fibrillar protein aggregation. Sci. Rep..

[33] Wojtas A.M., Dammer E.B., Guo Q., Ping L., Shantaraman A., Duong D.M., Yin L., Fox E.J., Seifar F., Lee E.B. (2024). Proteomic changes in the human cerebrovasculature in Alzheimer's disease and related tauopathies linked to peripheral biomarkers in plasma and cerebrospinal fluid. Alzheimer's Dement..

[34] Matsuda S., Matsuda Y., D'Adamio L. (2009). BRI3 inhibits amyloid precursor protein processing in a mechanistically distinct manner from its homologue dementia gene BRI2. J. Biol. Chem..

[35] Oien K.A., McGregor F., Butler S., Ferrier R.K., Downie I., Bryce S., Burns S., Keith W.N. (2004). Gastrokine 1 is abundantly and specifically expressed in superficial gastric epithelium, down-regulated in gastric carcinoma, and shows high evolutionary conservation. J. Pathol..

[36] Menheniott T.R., Kurklu B., Giraud A.S. (2013). Gastrokines: stomach-specific proteins with putative homeostatic and tumor suppressor roles. Am. J. Physiol. Gastrointest. Liver Physiol..

[37] Yoshikawa Y., Mukai H., Hino F., Asada K., Kato I. (2000). Isolation of two novel genes, down-regulated in gastric cancer. Jpn. J. Cancer Res..

[38] Martin T.E., Powell C.T., Wang Z., Bhattacharyya S., Walsh-Reitz M.M., Agarwal K., Toback F.G. (2003). A novel mitogenic protein that is highly expressed in cells of the gastric antrum mucosa. Am. J. Physiol. Gastrointest. Liver Physiol..

[39] Moss S.F., Lee J.W., Sabo E., Rubin A.K., Rommel J., Westley B.R., May F.E.B., Gao J., Meitner P.A., Tavares R., Resnick M.B. (2008). Decreased expression of gastrokine 1 and the trefoil factor interacting protein TFIZ1/GKN2 in gastric cancer: influence of tumor histology and relationship to prognosis. Clin. Cancer Res..

[40] Ouyang J., Pan X., Lin H., Hu Z., Xiao P., Hu H. (2017). GKN2 increases apoptosis, reduces the proliferation and invasion ability of gastric cancer cells through down-regulating the JAK/STAT signaling pathway. Am. J. Transl. Res..

[41] Menheniott T.R., O'Connor L., Chionh Y.T., Däbritz J., Scurr M., Rollo B.N., Ng G.Z., Jacobs S., Catubig A., Kurklu B. (2016). Loss of gastrokine-2 drives premalignant gastric inflammation and tumor progression. J. Clin. Investig..

[42] Kuai X., Li L., Chen R., Wang K., Chen M., Cui B., Zhang Y., Li J., Zhu H., Zhou H. (2019). SCF(FBXW7)/GSK3beta-Mediated GFI1 Degradation Suppresses Proliferation of Gastric Cancer Cells. Cancer Res..

[43] Rooney S.A., Young S.L., Mendelson C.R. (1994). Molecular and cellular processing of lung surfactant. FASEB J..

[44] Phelps D.S., Floros J. (1991). Localization of pulmonary surfactant proteins using immunohistochemistry and tissue in situ hybridization. Exp. Lung Res..

[45] Beers M.F., Fisher A.B. (1992). Surfactant protein C a review of its unique properties and metabolism. Am. J. Physiol..

[46] Willander H., Askarieh G., Landreh M., Westermark P., Nordling K., Keränen H., Hermansson E., Hamvas A., Nogee L.M., Bergman T. (2012). High-resolution structure of a BRICHOS domain and its implications for anti-amyloid chaperone activity on lung surfactant protein C. Proc. Natl. Acad. Sci. USA.

[47] Ghosh D., Torres F., Schneider M.M., Ashkinadze D., Kadavath H., Fleischmann Y., Mergenthal S., Güntert P., Krainer G., Andrzejewska E.A. (2024). The inhibitory action of the chaperone BRICHOS against the alpha-Synuclein secondary nucleation pathway. Nat. Commun..

[48] Arosio P., Michaels T.C.T., Linse S., Månsson C., Emanuelsson C., Presto J., Johansson J., Vendruscolo M., Dobson C.M., Knowles T.P.J. (2016). Kinetic analysis reveals the diversity of microscopic mechanisms through which molecular chaperones suppress amyloid formation. Nat. Commun..

[49] Hiraki Y., Tanaka H., Inoue H., Kondo J., Kamizono A., Suzuki F. (1991). Molecular cloning of a new class of cartilage-specific matrix, chondromodulin-I, which stimulates growth of cultured chondrocytes. Biochem. Biophys. Res. Commun..

[50] Zhu S., Qiu H., Bennett S., Kuek V., Rosen V., Xu H., Xu J. (2019). Chondromodulin-1 in health, osteoarthritis, cancer, and heart disease. Cell. Mol. Life Sci..

[51] Fujii M., Furumatsu T., Yokoyama Y., Kanazawa T., Kajiki Y., Abe N., Ozaki T. (2013). Chondromodulin-I derived from the inner meniscus prevents endothelial cell proliferation. J. Orthop. Res..

[52] Zhu Y., Zhang Y., Liu Y., Tao R., Xia H., Zheng R., Shi Y., Tang S., Zhang W., Liu W. (2015). The influence of Chm-I knockout on ectopic cartilage regeneration and homeostasis maintenance. Tissue Eng..

[53] Shukunami C., Hiraki Y. (2007). Chondromodulin-I and tenomodulin: the negative control of angiogenesis in connective tissue. Curr. Pharm. Des..

[54] Deng B., Chen C., Gong X., Guo L., Chen H., Yin L., Yang L., Wang F. (2017). ChondromodulinI expression and correlation with angiogenesis in human osteoarthritic cartilage. Mol. Med. Rep..

[55] Yoshioka M., Yuasa S., Matsumura K., Kimura K., Shiomi T., Kimura N., Shukunami C., Okada Y., Mukai M., Shin H. (2006). Chondromodulin-I maintains cardiac valvular function by preventing angiogenesis. Nat. Med..

[56] Shao J., Gan L., Wang J. (2016). Transfection of chondromodulin I into human breast cancer cells and its effect on the inhibition of cancer cell growth. Mol. Med. Rep..

[57] Lin X., Wang L., Wang F. (2017). ChondromodulinI suppresses tumorigenesis of human osteosarcoma cells. Mol. Med. Rep..

[58] Shukunami C., Oshima Y., Hiraki Y. (2001). Molecular cloning of tenomodulin, a novel chondromodulin-I related gene. Biochem. Biophys. Res. Commun..

[59] Brandau O., Meindl A., Fässler R., Aszódi A. (2001). A novel gene, tendin, is strongly expressed in tendons and ligaments and shows high homology with chondromodulin-I. Dev. Dyn..

[60] Lin D., Alberton P., Delgado Caceres M., Prein C., Clausen-Schaumann H., Dong J., Aszodi A., Shukunami C., Iatridis J.C., Docheva D. (2020). Loss of tenomodulin expression is a risk factor for age-related intervertebral disc degeneration. Aging Cell.

[61] Tolppanen A.M., Helisalmi S., Hiltunen M., Kolehmainen M., Schwab U., Pirttilä T., Pulkkinen L., Uusitupa M., Soininen H. (2011). Tenomodulin variants, APOE and Alzheimer's disease in a Finnish case-control cohort. Neurobiol. Aging.

[62] Tolppanen A.-M., Nevalainen T., Kolehmainen M., Seitsonen S., Immonen I., Uusitupa M., Kaarniranta K., Pulkkinen L. (2009). Single nucleotide polymorphisms of the tenomodulin gene (TNMD) in age-related macular degeneration. Mol. Vis..

[63] Pittois K., Wauters J., Bossuyt P., Deleersnijder W., Merregaert J. (1999). Genomic organization and chromosomal localization of the Itm2a gene. Mamm. Genome.

[64] Menheniott T.R., Peterson A.J., O'Connor L., Lee K.S., Kalantzis A., Kondova I., Bontrop R.E., Bell K.M., Giraud A.S. (2010). A novel gastrokine, Gkn3, marks gastric atrophy and shows evidence of adaptive gene loss in humans. Gastroenterology.

[65] Bockerstett K.A., Lewis S.A., Noto C.N., Ford E.L., Saenz J.B., Jackson N.M., Ahn T.H., Mills J.C., DiPaolo R.J. (2020). Single-Cell Transcriptional Analyses Identify Lineage-Specific Epithelial Responses to Inflammation and Metaplastic Development in the Gastric Corpus. Gastroenterology.

[66] Nerelius C., Gustafsson M., Nordling K., Larsson A., Johansson J. (2009). Anti-Amyloid Activity of the C-Terminal Domain of proSP-C against Amyloid β-Peptide and Medin. Biochemistry.

[67] Szyperski T., Vandenbussche G., Curstedt T., Curstedt T., Ruysschaert J.M., Wüthrich K., Johansson J. (1998). Pulmonary surfactant-associated polypeptide C in a mixed organic solvent transforms from a monomeric alpha-helical state into insoluble beta-sheet aggregates. Protein Sci..

[68] Gustafsson M., Thyberg J., Näslund J., Eliasson E., Johansson J. (1999). Amyloid fibril formation by pulmonary surfactant protein C. FEBS Lett..

[69] Kallberg Y., Gustafsson M., Persson B., Thyberg J., Johansson J. (2001). Prediction of amyloid fibril-forming proteins. J. Biol. Chem..

[70] Johansson J. (2024). Treatment with BRICHOS domain helps to clarify issues with Alzheimer mouse models. EMBO Mol. Med..

[71] Sun J., Feng H., Xing W., Han Y., Suo J., Yallowitz A.R., Qian N., Shi Y., Greenblatt M.B., Zou W. (2020). Histone demethylase LSD1 is critical for endochondral ossification during bone fracture healing. Sci. Adv..

[72] Bartel D.P. (2009). MicroRNAs: target recognition and regulatory functions. Cell.

[73] Wang K., Sun T., Li N., Wang Y., Wang J.X., Zhou L.Y., Long B., Liu C.Y., Liu F., Li P.F. (2014). MDRL lncRNA regulates the processing of miR-484 primary transcript by targeting miR-361. PLoS Genet..

[74] Wang K., Liu C.Y., Zhou L.Y., Wang J.X., Wang M., Zhao B., Zhao W.K., Xu S.J., Fan L.H., Zhang X.J. (2015). APF lncRNA regulates autophagy and myocardial infarction by targeting miR-188-3p. Nat. Commun..

[75] Chodurska B., Kunej T. (2025). Long non-coding RNAs in humans: Classification, genomic organization and function. Noncoding. RNA Res..

[76] Hansen T.B., Jensen T.I., Clausen B.H., Bramsen J.B., Finsen B., Damgaard C.K., Kjems J. (2013). Natural RNA circles function as efficient microRNA sponges. Nature.

[77] Fernandes J., Vieira A.S., Kramer-Soares J.C., Da Silva E.A., Lee K.S., Lopes-Cendes I., Arida R.M. (2018). Hippocampal microRNA-mRNA regulatory network is affected by physical exercise. Biochim. Biophys. Acta Gen. Subj..

[78] Ou D., Nie J., Liu Y., Zhang L., Wang X., Chen Y., Wang H., Gong L., Li Z., Marcucci F., Liu D. (2025). The potential of circITFG2 as a therapeutic target in lung squamous cell carcinoma. J. Thorac. Dis..

[79] Lambert S.A., Jolma A., Campitelli L.F., Das P.K., Yin Y., Albu M., Chen X., Taipale J., Hughes T.R., Weirauch M.T. (2018). The Human Transcription Factors. Cell.

[80] Tai T.S., Pai S.Y., Ho I.C. (2014). Itm2a, a target gene of GATA-3, plays a minimal role in regulating the development and function of T cells. PLoS One.

[81] Barber T.D., Barber M.C., Tomescu O., Barr F.G., Ruben S., Friedman T.B. (2002). Identification of target genes regulated by PAX3 and PAX3-FKHR in embryogenesis and alveolar rhabdomyosarcoma. Genomics.

[82] Guo Y.P., Zhi Y.R., Liu T.T., Wang Y., Zhang Y. (2019). Global Gene Knockout of Kcnip3 Enhances Pain Sensitivity and Exacerbates Negative Emotions in Rats. Front. Mol. Neurosci..

[83] Zhao N.Q., Pan C.Y., Zhang T.Z., Liu P., Hu T.Z., Shang Q., Luo H., Fang Q., Wang J.S. (2023). Study on the Relationship between Integrin 2A and Drug Resistance in Chronic Myeloid Leukemia. J. Exp. Hematol..

[84] Adekoya T.O., Smith N., Kothari P., Dacanay M.A., Li Y., Richardson R.M. (2024). CXCR1 Expression in MDA-PCa-2b Cell Upregulates ITM2A to Inhibit Tumor Growth. Cancers (Basel).

[85] Jiang J., Xu J., Ou L., Yin C., Wang Y., Shi B. (2024). ITM2A inhibits the progression of bladder cancer by downregulating the phosphorylation of STAT3. Am. J. Cancer Res..

[86] Fan J., Zhang X., Kang M., Lee C.S., Kim L., Hadaya D., Aghaloo T.L., Lee M. (2023). Complementary modulation of BMP signaling improves bone healing efficiency. Biomaterials.

[87] Zhu S., Chen W., Masson A., Li Y.P. (2024). Cell signaling and transcriptional regulation of osteoblast lineage commitment, differentiation, bone formation, and homeostasis. Cell Discov..

[88] Salhotra A., Shah H.N., Levi B., Longaker M.T. (2020). Mechanisms of bone development and repair. Nat. Rev. Mol. Cell Biol..

[89] Wu M., Wu S., Chen W., Li Y.P. (2024). The roles and regulatory mechanisms of TGF-beta and BMP signaling in bone and cartilage development, homeostasis and disease. Cell Res..

[90] Schindler C., Plumlee C. (2008). Inteferons pen the JAK-STAT pathway. Semin. Cell Dev. Biol..

[91] Obeagu E.I. (2025). JAK2 in pediatric leukemia: mechanisms of pathogenesis and drug development - a narrative review. Ann. Med. Surg..

[92] Hu Q., Bian Q., Rong D., Wang L., Song J., Huang H.S., Zeng J., Mei J., Wang P.Y. (2023). JAK/STAT pathway: Extracellular signals, diseases, immunity, and therapeutic regimens. Front. Bioeng. Biotechnol..

[93] Hu X., Li J., Fu M., Zhao X., Wang W. (2021). The JAK/STAT signaling pathway: from bench to clinic. Signal Transduct. Targeted Ther..

[94] Peyssonnaux C., Eychène A. (2001). The Raf/MEK/ERK pathway: new concepts of activation. Biol. Cell.

[95] Nasir N.J.N., Arifin N., Noordin K.B.A.A., Yusop N. (2023). Bone repair and key signalling pathways for cell-based bone regenerative therapy: A review. J. Taibah Univ. Med. Sci..

[96] Kim J.M., Yang Y.S., Hong J., Chaugule S., Chun H., van der Meulen M.C., Xu R., Greenblatt M.B., Shim J.h. (2022). Biphasic regulation of osteoblast development via the ERK MAPK-mTOR pathway. eLife.

[97] Dhillon A.S., Hagan S., Rath O., Kolch W. (2007). MAP kinase signalling pathways in cancer. Oncogene.

[98] Guo Y.J., Pan W.W., Liu S.B., Shen Z.F., Xu Y., Hu L.L. (2020). ERK/MAPK signalling pathway and tumorigenesis. Exp. Ther. Med..

[99] Zhuang T. (2025). Hedgehog pathway, cell cycle, and primary cilium. Cell Death Discov..

[100] Gorojankina T. (2016). Hedgehog signaling pathway: a novel model and molecular mechanisms of signal transduction. Cell. Mol. Life Sci..

[101] Cong G., Zhu X., Chen X.R., Chen H., Chong W. (2025). Mechanisms and therapeutic potential of the hedgehog signaling pathway in cancer. Cell Death Discov..

[102] Morales-Alcala C.C., Georgiou I.C., Timmis A.J., Riobo-Del Galdo N.A. (2021). Integral Membrane Protein 2A Is a Negative Regulator of Canonical and Non-Canonical Hedgehog Signalling. Cells.

[103] Liu Z.X., Pi W.L., Dai X.L., Huo Q., Zuo Z.P., Sun Y.X. (2025). Role of the cAMP-PKA-CREB pathway in depression: mechanisms and therapeutic implications. Brain Res..

[104] Ahmed M.B., Alghamdi A.A.A., Islam S.U., Lee J.S., Lee Y.S. (2022). cAMP Signaling in Cancer: A PKA-CREB and EPAC-Centric Approach. Cells.

[105] Zhang H., Kong Q., Wang J., Jiang Y., Hua H. (2020). Complex roles of cAMP-PKA-CREB signaling in cancer. Exp. Hematol. Oncol..

[106] Taylor S.S., Ilouz R., Zhang P., Kornev A.P. (2012). Assembly of allosteric macromolecular switches: lessons from PKA. Nat. Rev. Mol. Cell Biol..

[107] Kovall R.A., Gebelein B., Sprinzak D., Kopan R. (2017). The Canonical Notch Signaling Pathway: Structural and Biochemical Insights into Shape, Sugar, and Force. Dev. Cell.

[108] Siebel C., Lendahl U. (2017). Notch Signaling in Development, Tissue Homeostasis, and Disease. Physiol. Rev..

[109] Aster J.C., Pear W.S., Blacklow S.C. (2017). The Varied Roles of Notch in Cancer. Annu. Rev. Pathol..

[110] Saxton R.A., Sabatini D.M. (2017). mTOR Signaling in Growth, Metabolism, and Disease. Cell.

[111] Liu G.Y., Sabatini D.M. (2020). mTOR at the nexus of nutrition, growth, ageing and disease. Nat. Rev. Mol. Cell Biol..

[112] Rinne N., Christie E.L., Ardasheva A., Kwok C.H., Demchenko N., Low C., Tralau-Stewart C., Fotopoulou C., Cunnea P. (2021). Targeting the PI3K/AKT/mTOR pathway in epithelial ovarian cancer, therapeutic treatment options for platinum-resistant ovarian cancer. Cancer Drug Resist..

[113] Van den Plas D., Merregaert J. (2004). Constitutive overexpression of the integral membrane protein Itm2A enhances myogenic differentiation of C2C12 cells. Cell Biol. Int..

[114] Long F., Ornitz D.M. (2013). Development of the endochondral skeleton. Cold Spring Harbor Perspect. Biol..

[115] Tuckermann J.P., Pittois K., Partridge N.C., Merregaert J., Angel P. (2000). Collagenase-3 (MMP-13) and Integral Membrane Protein 2a (Itm2a) are Marker Genes of Chondrogenic/Osteoblastic Cells in Bone Formation: Sequential Temporal, and Spatial Expression of Itm2a, Alkaline Phosphatase, MMP-13, and Osteocalcin in the Mouse. J. Bone Miner. Res..

[116] Sirola K. (1960). Regeneration of defects in the calvaria. An experimental study. Ann. Med. Exp. Biol. Fenn..

[117] Aalami O.O., Nacamuli R.P., Lenton K.A., Cowan C.M., Fang T.D., Fong K.D., Shi Y.Y., Song H.M., Sahar D.E., Longaker M.T. (2004). Applications of a Mouse Model of Calvarial Healing: Differences in Regenerative Abilities of Juveniles and Adults. Plast. Reconstr. Surg..

[118] Hobar P.C., Masson J.A., Wilson R., Zerwekh J. (1996). The Importance of the Dura in Craniofacial Surgery. Plast. Reconstr. Surg..

[119] Wan D.C., Aalami O.O., Wang Z., Nacamuli R.P., Lorget F., Derynck R., Longaker M.T. (2006). Differential gene expression between juvenile and adult dura mater: a window into what genes play a role in the regeneration of membranous bone. Plast. Reconstr. Surg..

[120] Wilson A., Shehadeh L.A., Yu H., Webster K.A. (2010). Age-related molecular genetic changes of murine bone marrow mesenchymal stem cells. BMC Genom..

[121] Castagné R., Zeller T., Rotival M., Szymczak S., Truong V., Schillert A., Trégouët D.A., Münzel T., Ziegler A., Cambien F. (2011). Influence of sex and genetic variability on expression of X-linked genes in human monocytes. Genomics.

[122] Tukiainen T., Pirinen M., Sarin A.P., Ladenvall C., Kettunen J., Lehtimäki T., Lokki M.L., Perola M., Sinisalo J., Vlachopoulou E. (2014). Chromosome X-wide association study identifies Loci for fasting insulin and height and evidence for incomplete dosage compensation. PLoS Genet..

[123] Moerman E.J., Teng K., Lipschitz D.A., Lecka-Czernik B. (2004). Aging activates adipogenic and suppresses osteogenic programs in mesenchymal marrow stroma/stem cells: the role of PPAR-gamma2 transcription factor and TGF-beta/BMP signaling pathways. Aging Cell.

[124] Nishida S., Endo N., Yamagiwa H., Tanizawa T., Takahashi H.E. (1999). Number of osteoprogenitor cells in human bone marrow markedly decreases after skeletal maturation. J. Bone Miner. Metabol..

[125] Zheng H., Martin J.A., Duwayri Y., Falcon G., Buckwalter J.A. (2007). Impact of Aging on Rat Bone Marrow-Derived Stem Cell Chondrogenesis. J. Gerontol. A Biol. Sci. Med. Sci..

[126] Chen L., Hassani Nia F., Stauber T. (2021). Ion Channels and Transporters in Muscle Cell Differentiation. Int. J. Mol. Sci..

[127] Kim K.M., Jang H.C., Lim S. (2016). Differences among skeletal muscle mass indices derived from height-weight-and body mass index-adjusted models in assessing sarcopenia. Korean J. Intern. Med. (Engl. Ed.).

[128] Lee E.J., Jan A.T., Baig M.H., Ashraf J.M., Nahm S.S., Kim Y.W., Park S.Y., Choi I. (2016). Fibromodulin: a master regulator of myostatin controlling progression of satellite cells through a myogenic program. FASEB J..

[129] Yamakawa H., Kusumoto D., Hashimoto H., Yuasa S. (2020). Stem Cell Aging in Skeletal Muscle Regeneration and Disease. Int. J. Mol. Sci..

[130] Cossu G., Biressi S. (2005). Satellite cells, myoblasts and other occasional myogenic progenitors: possible origin, phenotypic features and role in muscle regeneration. Semin. Cell Dev. Biol..

[131] Williamson D.L., Butler D.C., Alway S.E. (2009). AMPK inhibits myoblast differentiation through a PGC-1alpha-dependent mechanism. Am. J. Physiol. Endocrinol. Metab..

[132] Franklin D.S., Xiong Y. (1996). Induction of p18INK4c and its predominant association with CDK4 and CDK6 during myogenic differentiation. Mol. Biol. Cell.

[133] Lee E.J., Jan A.T., Baig M.H., Ahmad K., Malik A., Rabbani G., Kim T., Lee I.K., Lee Y.H., Park S.Y., Choi I. (2018). Fibromodulin and regulation of the intricate balance between myoblast differentiation to myocytes or adipocyte-like cells. FASEB J..

[134] Gerber A.N., Wilson C.W., Li Y.J., Chuang P.T. (2007). The hedgehog regulated oncogenes Gli1 and Gli2 block myoblast differentiation by inhibiting MyoD-mediated transcriptional activation. Oncogene.

[135] Koleva M., Kappler R., Vogler M., Herwig A., Fulda S., Hahn H. (2005). Pleiotropic effects of sonic hedgehog on muscle satellite cells. Cell. Mol. Life Sci..

[136] Lim J.H., Beg M.M.A., Ahmad K., Shaikh S., Ahmad S.S., Chun H.J., Choi D., Lee W.J., Jin J.O., Kim J. (2021). IgLON5 Regulates the Adhesion and Differentiation of Myoblasts. Cells.

[137] Yin H., Price F., Rudnicki M.A. (2013). Satellite cells and the muscle stem cell niche. Physiol. Rev..

[138] Boyko A.R., Brooks S.A., Behan-Braman A., Castelhano M., Corey E., Oliveira K.C., Swinburne J.E., Todhunter R.J., Zhang Z., Ainsworth D.M., Robinson N.E. (2014). Genomic analysis establishes correlation between growth and laryngeal neuropathy in Thoroughbreds. BMC Genom..

[139] Kihara M., Kiyoshima T., Nagata K., Wada H., Fujiwara H., Hasegawa K., Someya H., Takahashi I., Sakai H. (2014). Itm2a expression in the developing mouse first lower molar, and the subcellular localization of Itm2a in mouse dental epithelial cells. PLoS One.

[140] Obermeier B., Daneman R., Ransohoff R.M. (2013). Development, maintenance and disruption of the blood-brain barrier. Nat. Med..

[141] Cegarra C., Chaves C., Déon C., Do T.M., Dumas B., Frenzel A., Kuhn P., Roudieres V., Guillemot J.C., Lesuisse D. (2022). Exploring ITM2A as a new potential target for brain delivery. Fluids Barriers CNS.

[142] Banks W.A. (2016). From blood–brain barrier to blood–brain interface: new opportunities for CNS drug delivery. Nat. Rev. Drug Discov..

[143] Bangsow T., Baumann E., Bangsow C., Jaeger M.H., Pelzer B., Gruhn P., Wolf S., von Melchner H., Stanimirovic D.B. (2008). The epithelial membrane protein 1 is a novel tight junction protein of the blood-brain barrier. J. Cereb. Blood Flow Metab..

[144] Shusta E.V., Boado R.J., Mathern G.W., Pardridge W.M. (2002). Vascular Genomics of the Human Brain. J. Cereb. Blood Flow Metab..

[145] Feng W., Chen L., Nguyen P.K., Wu S.M., Li G. (2019). Single Cell Analysis of Endothelial Cells Identified Organ-Specific Molecular Signatures and Heart-Specific Cell Populations and Molecular Features. Front. Cardiovasc. Med..

[146] Pardridge W.M. (2007). Blood-brain barrier genomics. Stroke.

[147] Li J.Y., Boado R.J., Pardridge W.M. (2002). Rat Blood–Brain Barrier Genomics. II. J. Cereb. Blood Flow Metab..

[148] Takács L., Tóth E., Losonczy G., Szanto A., Bähr-Ivacevic T., Benes V., Berta A., Vereb G. (2011). Differentially expressed genes associated with human limbal epithelial phenotypes: new molecules that potentially facilitate selection of stem cell-enriched populations. Investig. Ophthalmol. Vis. Sci..

[149] Kirchner J., Bevan M.J. (1999). ITM2A is induced during thymocyte selection and T cell activation and causes downregulation of CD8 when overexpressed in CD4(+)CD8(+) double positive thymocytes. J. Exp. Med..

[150] Kulus M., Sujka-Kordowska P., Konwerska A., Celichowski P., Kranc W., Kulus J., Piotrowska-Kempisty H., Antosik P., Bukowska D., Iżycki D. (2019). New Molecular Markers Involved in Regulation of Ovarian Granulosa Cell Morphogenesis, Development and Differentiation during Short-Term Primary In Vitro Culture-Transcriptomic and Histochemical Study Based on Ovaries and Individual Separated Follicles. Int. J. Mol. Sci..

[151] Zhang R., Xu T., Xia Y., Wang Z., Li X., Chen W. (2021). ITM2A as a Tumor Suppressor and Its Correlation With PD-L1 in Breast Cancer. Front. Oncol..

[152] Abuderman A.A., Harb O.A., Gertallah L.M. (2020). Prognostic and clinicopathological values of tissue expression of MFAP5 and ITM2A in triple-negative breast cancer: an immunohistochemical study. Contemp. Oncol..

[153] Jiang C., Feng R., Zhao Y., Zhang J., Han N., Zhang Y., Shu G., Yin G., Wang M. (2025). ITM2A as a potential prognostic marker for triple-negative breast cancer. J. Cancer.

[154] Saha S., Panigrahi D.P., Patil S., Bhutia S.K. (2018). Autophagy in health and disease: A comprehensive review. Biomed. Pharmacother..

[155] Duraes F.V., Niven J., Dubrot J., Hugues S., Gannagé M. (2015). Macroautophagy in Endogenous Processing of Self- and Pathogen-Derived Antigens for MHC Class II Presentation. Front. Immunol..

[156] Yin H., Zhao L., Li S., Xu L., Wang Y., Chen H. (2017). Impaired Cellular Energy Metabolism Contributes to Duck-Enteritis-Virus-Induced Autophagy via the AMPK-TSC2-MTOR Signaling Pathway. Front. Cell. Infect. Microbiol..

[157] Kim J., Kundu M., Viollet B., Guan K.L. (2011). AMPK and mTOR regulate autophagy through direct phosphorylation of Ulk1. Nat. Cell Biol..

[158] Xue J.-F., Shi Z.-M., Zou J., Li X.L. (2017). Inhibition of PI3K/AKT/mTOR signaling pathway promotes autophagy of articular chondrocytes and attenuates inflammatory response in rats with osteoarthritis. Biomed. Pharmacother..

[159] Jin S., Gao J., Qi Y., Hao Y., Li X., Liu Q., Liu J., Liu D., Zhu L., Lin B. (2020). TGF-β1 fucosylation enhances the autophagy and mitophagy via PI3K/Akt and Ras-Raf-MEK-ERK in ovarian carcinoma. Biochem. Biophys. Res. Commun..

[160] Racacho K.J., Shiau Y.P., Villa R., Mahri S., Tang M., Lin T.Y., Li Y. (2025). The tumor immune microenvironment: implications for cancer immunotherapy, treatment strategies, and monitoring approaches. Front. Immunol..

[161] Bray F., Ferlay J., Soerjomataram I., Siegel R.L., Torre L.A., Jemal A. (2018). Global cancer statistics 2018: GLOBOCAN estimates of incidence and mortality worldwide for 36 cancers in 185 countries. CA Cancer J. Clin..

[162] Seng L.M., Rosman A.N., Khan A., Haris N.M., Mustapha N.A.S., Husaini N.S.M., Zahari N.F. (2018). Awareness of cervical cancer among women in Malaysia. Int. J. Health Sci..

[163] Zhao M., Huang W., Zou S., Shen Q., Zhu X. (2020). A Five-Genes-Based Prognostic Signature for Cervical Cancer Overall Survival Prediction. Int. J. Genomics.

[164] Meng H., Liu J., Qiu J., Nie S., Jiang Y., Wan Y., Cheng W. (2020). Identification of Key Genes in Association with Progression and Prognosis in Cervical Squamous Cell Carcinoma. DNA Cell Biol..

[165] Davidson B., Abeler V.M., Hellesylt E., Holth A., Shih I.M., Skeie-Jensen T., Chen L., Yang Y., Wang T.L. (2013). Gene expression signatures differentiate uterine endometrial stromal sarcoma from leiomyosarcoma. Gynecol. Oncol..

[166] Zhang Z., Li J., He T., Ouyang Y., Huang Y., Liu Q., Wang P., Ding J. (2019). The competitive endogenous RNA regulatory network reveals potential prognostic biomarkers for overall survival in hepatocellular carcinoma. Cancer Sci..

[167] Zhou Q., Tao C., Ge Y., Yuan J., Pan F., Lin X., Wang R. (2024). A novel single-cell model reveals ferroptosis-associated biomarkers for individualized therapy and prognostic prediction in hepatocellular carcinoma. BMC Biol..

[168] Wang X., Chan S., Dai L., Xu Y., Yang Q., Wang M., Han Q., Chen J., Zuo X., Wang Z. (2024). Identification of novel T cell proliferation patterns, potential biomarkers and therapeutic drugs in colorectal cancer. J. Cancer.

[169] Wang S., Wu P., Xu N., Xiao Z., Liu Y., Bian W., Meng L., Guo R., Xu Y., Ding H. (2025). MSR1 + macrophages passivate antitumor immunity by inducing ITM2A + CD4T exhaustion differentiation. Hepatology.

[170] Zeng X., Yang D., Zhang J., Li K., Wang X., Ma F., Liao X., Wang Z., Zeng X., Zhang P. (2024). Integrating machine learning, bioinformatics and experimental verification to identify a novel prognostic marker associated with tumor immune microenvironment in head and neck squamous carcinoma. Front. Immunol..

[171] Cao Z., Ao Y., Guo Y., Zhou S. (2020). Comprehensive Analysis of mRNA Expression Profiles in Head and Neck Cancer by Using Robust Rank Aggregation and Weighted Gene Coexpression Network Analysis. Biomed Res. Int..

[172] Ou L. (2021).

[173] Buyuk B., Jin S., Ye K. (2022). Epithelial-to-Mesenchymal Transition Signaling Pathways Responsible for Breast Cancer Metastasis. Cell. Mol. Bioeng..

[174] Nagy Á., Ősz Á., Budczies J., Krizsán S., Szombath G., Demeter J., Bödör C., Győrffy B. (2019). Elevated HOX gene expression in acute myeloid leukemia is associated with NPM1 mutations and poor survival. J. Adv. Res..

[175] Hou Q., Liao F., Zhang S., Zhang D., Zhang Y., Zhou X., Xia X., Ye Y., Yang H., Li Z. (2017). Regulatory network of GATA3 in pediatric acute lymphoblastic leukemia. Oncotarget.

[176] Xia S.J., Pressey J.G., Barr F.G. (2002). Molecular pathogenesis of rhabdomyosarcoma. Cancer Biol. Ther..

[177] Giordano M., Gertosio C., Pagani S., Meazza C., Fusco I., Bozzola E., Bozzola M. (2015). A 5.8 Mb interstitial deletion on chromosome Xq21.1 in a boy with intellectual disability, cleft palate, hearing impairment and combined growth hormone deficiency. BMC Med. Genet..

[178] Sandell L.J., Aigner T. (2001). Articular cartilage and changes in arthritis. An introduction: cell biology of osteoarthritis. Arthritis Res. Ther..

[179] Steck E., Boeuf S., Gabler J., Werth N., Schnatzer P., Diederichs S., Richter W. (2012). Regulation of H19 and its encoded microRNA-675 in osteoarthritis and under anabolic and catabolic in vitro conditions. J. Mol. Med..

[180] Taurog J.D., Chhabra A., Colbert R.A. (2016). Ankylosing Spondylitis and Axial Spondyloarthritis. N. Engl. J. Med..

[181] Lari A., Gholami Pourbadie H., Jafari M., Sharifi-Zarchi A., Akhtari M., Nejatbakhsh Samimi L., Jamshidi A., Mahmoudi M. (2021). Downregulation of ITM2A Gene Expression in Macrophages of Patients with Ankylosing Spondylitis. Int. Arch. Allergy Immunol..

[182] Lee Y.H., Rho Y.H., Choi S.J., Ji J.D., Song G.G. (2005). Ankylosing spondylitis susceptibility loci defined by genome-search meta-analysis. J. Hum. Genet..

[183] Lee Y.H., Song G.G. (2015). Meta-analysis of differentially expressed genes in ankylosing spondylitis. Genet. Mol. Res..

[184] AlEjielat R., Khaleel A., Tarkhan A.H. (2021). Differential gene expression analysis of ankylosing spondylitis shows deregulation of the HLA-DRB, HLA-DQB, ITM2A, and CTLA4 genes. Egypt. J. Med. Hum. Genet..

[185] Stassi G., De Maria R. (2002). Autoimmune thyroid disease: new models of cell death in autoimmunity. Nat. Rev. Immunol..

[186] Menconi F., Marcocci C., Marinò M. (2014). Diagnosis and classification of Graves' disease. Autoimmun. Rev..

[187] Lockshin M.D. (2006). Sex differences in autoimmune disease. Lupus.

[188] Ishido N., Inoue N., Watanabe M., Hidaka Y., Iwatani Y. (2015). The relationship between skewed X chromosome inactivation and the prognosis of Graves' and Hashimoto's diseases. Thyroid.

[189] Libert C., Dejager L., Pinheiro I. (2010). The X chromosome in immune functions: when a chromosome makes the difference. Nat. Rev. Immunol..

[190] Yin X., Latif R., Tomer Y., Davies T.F. (2007). Thyroid epigenetics: X chromosome inactivation in patients with autoimmune thyroid disease. Ann. N. Y. Acad. Sci..

[191] Regitz-Zagrosek V., Kararigas G. (2017). Mechanistic Pathways of Sex Differences in Cardiovascular Disease. Physiol. Rev..

[192] Mosbah H., Donadille B., Vatier C., Janmaat S., Atlan M., Badens C., Barat P., Béliard S., Beltrand J., Ben Yaou R. (2022). Dunnigan lipodystrophy syndrome: French National Diagnosis and Care Protocol (PNDS; Protocole National de Diagnostic et de Soins). Orphanet J. Rare Dis..

[193] Davies S.J., Ryan J., O'Connor P.B.F., Kenny E., Morris D., Baranov P.V., O'Connor R., McCarthy T.V. (2017). Itm2a silencing rescues lamin A mediated inhibition of 3T3-L1 adipocyte differentiation. Adipocyte.

[194] Patil J., Lande J.D., Li N., Berryman T.R., King R.A., Hertz M.I. (2008). Bronchoalveolar lavage cell gene expression in acute lung rejection: development of a diagnostic classifier. Transplantation.

[195] Halloran P.F., Venner J.M., Madill-Thomsen K.S., Einecke G., Parkes M.D., Hidalgo L.G., Famulski K.S. (2018). Review: The transcripts associated with organ allograft rejection. Am. J. Transplant..

[196] Bonaccorsi-Riani E., Pennycuick A., Londoño M.C., Lozano J.J., Benítez C., Sawitzki B., Martínez-Picola M., Bohne F., Martínez-Llordella M., Miquel R. (2016). Molecular Characterization of Acute Cellular Rejection Occurring During Intentional Immunosuppression Withdrawal in Liver Transplantation. Am. J. Transplant..

[197] Mwanza J.C., Kahindo A.K., Mbusa-Kombi J., Mumbere M.V., Kitenge R.O., McIlwain D.R., Mulangu J.C.S., Mbala P.K., Okitundu D., Giordani B.J. (2025). Ophthalmological manifestations and plasma markers of inflammation in Ebola survivors in post-treatment era. Sci. Rep..

[198] Li Y.J., Zhang X.Y., Zhang W.J., Han Y.L., Li M.S., Zhao J.L., Wu J., Li X.W., Xu J., Shi F.D. (2024). Proteomics analysis of immune response-related proteins in Guillain-Barré Syndrome (GBS) and Chronic Inflammatory Demyelinating Polyneuropathy (CIDP). J. Neuroimmunol..

